# Supplementary material for: Prevalence and risk factors associated with asymptomatic Plasmodium falciparum infection and anemia among pregnant women at the first antenatal care visit: A hospital based cross-sectional study in Kwale County, Kenya
Source: PLoS One. 2020 Oct 8;15(10):e0239578. doi: 10.1371/journal.pone.0239578 (PMC7544053; doi:10.1371/journal.pone.0239578)
Supplement: Attachment — Submitted filename: PONE-D-20-11384_R1_reviewer-1.pdf [file pone.0239578.s002.pdf]

PLOS ONE

Prevalence and risk factors associated with asymptomatic Plasmodium falciparum infection and anemia among pregnant women at the first antenatal care visit: A hospital based cross-sectional study in Kwale County, Kenya.

--Manuscript Draft--

**Manuscript Number:**	PONE-D-20-11384R1
**Article Type:**	Research Article
**Full Title:**	Prevalence and risk factors associated with asymptomatic Plasmodium falciparum infection and anemia among pregnant women at the first antenatal care visit: A hospital based cross-sectional study in Kwale County, Kenya.
**Short Title:**	Prevalence and risk factors associated with asymptomatic malaria and anemia among pregnant women
**Corresponding Author:**	Gibson Waweru Nyamu, Masters<br>Msambweni Referral Hospital<br>Kirinyaga, Kirinyaga KENYA
**Keywords:**	Anemia, Asymptomatic malaria, Pregnant, Kenya.
**Abstract:**	Background: Prevalence of both asymptomatic and symptomatic malaria in pregnancy (MiP) in Kenya ranges from 9% to 18%. We estimated the prevalence and factors associated with MiP and anemia in pregnancy (AiP) among asymptomatic women attending antenatal care (ANC) visits.<br>Methods: We performed a cross-sectional study among asymptomatic pregnant women attending ANC at Msambweni Hospital, between September 2018 and February 2019. Data was collected and analyzed in Epi Info 7. Descriptive statistics were calculated and we compared MiP and AiP in asymptomatic cases to those without either condition. Adjusted prevalence Odds ratios (aPOR) and 95% confidence intervals (CI) were calculated to identify factors associated with asymptomatic MiP and AiP.<br>Results: We interviewed 308 study participants; their mean age was 26.6 years (± 5.8 years), mean gestational age was 21.8 weeks (± 6.0 weeks), 173 (56.2%) were in the second trimester of pregnancy, 12.9 % (40/308) had MiP and 62.7 % had AiP. Women who were aged ≤ 20 years had three times likelihood of developing MiP (aPOR= 3.1 CI: 1.3 - 7.35) compared to those aged >20 years old. The likelihood of AiP was higher among women with gestational age ≥ 16 weeks (aPOR=3.9, CI: 1.96 – 7.75), those with parasitemia (aPOR= 3.3, 95% CI: 1.31-8.18), those in third trimester of pregnancy (aPOR= 2.6, 95% CI:1.40 - 4.96) and those who reported eating soil as a craving during pregnancy (aPOR=1.9, 95%CI:1.15 - 3.29)..<br>Conclusions: Majority of the women had asymptomatic AiP and MiP was observed in one tenth of all study participants. Asymptomatic MiP was associated with younger age while AiP was associated with gestational age parasitemia, and soil consumption as a craving during pregnancy.
**Order of Authors:**	Gibson Waweru Nyamu, Masters
	Elvis Oyugi Oyugi
	Hajara Busaidy Busaidy
	Jimmy Kihara Hussein
	Victor Omballa Omballa
	Victor Jeza Tunje
**Response to Reviewers:**	Reviewer #1<br>Questions/ConcernsComments Corrections<br>Major comments<br>Concern 1Abstract results: make it clear whether the results presented are adjusted odds ratios throughout.Corrected done it is adjusted prevalence odds ratios that has been used throughout the abstract<br>Concern 2Risk factors for AIP: The authors need to better explain how anaemia risk is

	apparently associated with BOTH first trimester, AND gestation >16 weeks. There are problems with results presentation in Table 3 and it seems they have got the reference and comparator groups switched for the gestation comparison. In addition, part of the results in the table are presented as percentages of women with risk factor who are anaemia (the table column heading) and part are presented as percentage of anaemic women who fall into each risk factor category (not what we want). Please redo this table and recheck your AORs and revise text accordingly.Yes, I have reanalyzed the anemia data, by use of backward elimination method, there seemed an interaction between trimester's variable and gestational variable. By removing trimester variable for it had a bigger p value, gestational >16 weeks resulted to AOR; 3.3 (1.72-6.41). I have changed the comparison groups accordingly both in the table and in the text<br>Concern 3. Discussion: differences in malaria prevalence between Kenya and Burkina Faso are more likely related to malaria transmission intensity, prevalence falls with gestation, and falls more in women receiving IPTp. (Related to this, please also discuss role of IPTp in controlling MIP in conclusions).Interventions measures for first visit pregnant women at antenatal clinic for malaria in pregnancy are; IPTp and provision of LLINs among others [17]. Although the role of IPTp is known to reduce maternal malaria episodes and improve pregnancy outcomes [33], the current study did not include pregnant women who had taken antimalarials hence we could not ascertain the role played by IPTp and majority of our study participants (82.7%) in the current study reported to always using bednet, whereas the study in Burkina Faso included pregnant women seeking ANC services at any point in their pregnancies. This may be factors in the difference between these two reported prevalence.<br><br>Concern 4Similarly, end P 11, haemodilution over pregnancy seems more likely to explain declining hb over pregnancy. If postulating dfetal needs please provide reference.<br>Anemia is a function of plasma volume and red cell mass; both of which increase during pregnancy; but the increase in plasma volume is proportionately greater than the increase in red cell mass [40]. Explanation has been given<br>Minor comments<br>Concern 1Abstract: line 1 "pregnancy". Results 3rd sentence rewrite " Women who had MIP were 12.9%". Last line of results: rewrite for clarity.<br>Corrected; pregnant to pregnancy<br>Rewritten; Women who had plasmodium infections were 12.9 % (40/308)<br><br>Concern 2There were a lot of spacing issues in the pdf, e.g. words joined together, or words and brackets without spaces before them. Examples in abstract methods: odds ratios(OR) and confidence intervals(CI)- there are many others.Attention to details in spacing has been addressed throughout the manuscript<br>Concern 3Introduction 3rd sentence change "malaria with AIP and consumption of soil (geophagy)", needs different punctuation?<br>It has been re-written<br>Concern 4. Next sentence: is this risk factors for MIP (parasitic infections? High gravidity is NOT associated with MIP) Or AIP?<br>It has been re-written<br>Concern 5<br><br>"In sub-Saharan Africa, MIP affects approximately 125 million pregnant women every year"- this is a major misreading of the cited reference.<br>It has been re-written<br><br>Concern 6Top of P 5 "a cross sectional"<br>It has been re- written<br>Concern7<br>Inclusion/exclusion: "as well as those who"<br>It has been re- written<br>Concern 8<br>Concern 8Sample size "desired level" of what was 5%?<br>Two-sided significance level defined at 5%<br><br>Concern 9Data management " between a variety…"<br>It has been re-written<br>Concern 10Page 8: "prevalence and risk factors for malaria parasitemia". Latent

malaria is not a recognised phrase. Next line remove “who”.
Latent replaced by asymptomatic and re-written
Concern 11 Parasite counts are best expressed as geometric mean not median.Geometric mean was 3738
Concern 12. P 9 defines mild, moderate, severe anaemia.
Mild, moderate and severe anemia has been defined in definition of terms
Concern 13Page 11 first paragraph contains multiple grammatical errors, please rewrite
It has been re looked and corrected accordingly
Concern 14Third paragraph same page please rewrite.It has been re looked and corrected accordingly
Concern 15A lot of the references are incomplete (lacking volumes/pages), or not enough details are given to retrieve them, or have formatting issues. Refs 2, 5, 6 (what is MOH? Where can this be obtained- similar comments for some others), 7, 17-22, 33.

The references has been corrected and the links are provided where necessary
Concern 16Table groups need editing to ensure e.g. it is clear which group women with 8 y school fall into. Same for gestation. And make table 2 N column wider so numbers are readable.
The table 2; corrected < 8 year in schooling and gestational age in weeks >16
It has been expanded and fonts increased for readability

Reviwer #2

Comments /questions Corrections/ responses
Concern 1Suggested title: “Prevalence and risk factors associated with asymptomatic Plasmodium falciparum infection and anaemia among pregnant women at the first antenatal care visit: A hospital based cross-sectional study in Kwale County, Kenya.
Use asymptomatic malaria is less specificPlasmodium falciparum was the predominant species more than 80% hence we concur with the reviewer
Prevalence and risk factors associated with asymptomatic Plasmodium falciparum infection and anemia among pregnant women at the first antenatal care visit: A hospital based cross-sectional study in Kwale County, Kenya.

Concern 2Abbreviations: Malaria in pregnancy (MiP and not MIP), Anaemia in pregnancy (AiP and not AIP)Have corrected the whole manuscript where applicable MIP to MiP and AIP to AiP
Concern 3Abstract:
a) Background sub-session: Prevalence of malaria in pregnancy, not … in pregnantCorrected to read Prevalence of malaria in pregnancy, not … in pregnant

b) Results sub-session: please specify what type of odds you used for AiP (crude or adjusted)We have corrected the omission, the odds Ratio was Adjusted Odd Ratio

c) Key words: important key words do not appear. Please use “asymptomatic malaria” instead of “parasitaemia” which could lead confusion as including symptomatic and asymptomatic casesHave replaced “Parasitaemia”, with “asymptomatic malaria”
Concern 4Introduction
a. The authors should provide more detail on the current policies against malaria and anaemia during pregnancy used in Kenya, particularly IPTp administration and timeline, iron and folate supplementation? Who in charge of these measures (Government or pregnant women themselves)?
“We have included these paragraphs
The focused antenatal care is a package given to pregnant women which entails giving prophylaxis and treatment for anemia and malaria, among other services, where the AiP is prevented by providing nutritional counseling including iron supplements, and treating cases of AiP [20].
An intervention aimed at prevention and control MiP adopted by the Ministry of Health (MOH) Kenya, are; Intermittent Preventive Treatment (IPTp) of MiP, with Sulfadoxine Pyrimethamine (SP) given after 12 weeks gestational period done four weeks apart until the pregnant woman delivers, in areas with high malaria transmission such as Western, Nyanza and Coast regions and Long-lasting Insecticidal Nets (LLINs) at the ANC in the first contact among others [21]

Powered by Editorial Manager® and ProduXion Manager® from Aries Systems Corporation

Page 3, line 6: “Other factors associated with MiP are……” Use “Other” supposes that you have cited first factors which are not the case. Please rephrase the sentence to make it clear.Besides plasmodium infections other factors known to cause AiP include nutritional deficiencies, infectious diseases like HIV, parasitic infections like hookworm infestation, and the hemoglobinopathies [4, 5].

c. Page 3, line 7: The authors state that “high parity” is associated with malaria. What do
you mean by high parity? Multigravidae women? If yes, I think it’s a wording mistake as it’s well-known that is the primigravidae who are higher risk
of MiP, so ‘less parity”.
Corrected; Several studies has documented factors associated with MiP are women with less parity,

d. The rational of the study is unclear as presented. Please, give more details explaining what the study brings for the scientific community which are not already known. The only fact that “factors contributing to MiP are not well-described in this part of Kenya” is not sufficient.
Kenya Malaria Indicator Survey (KMIS) 2015 reported 2 folds increase in the prevalence of malaria in the coastal region compared to KMIS 2010 [18]. There are a possibility plasmodium infections in healthy adults, including pregnant women, in moderate to high transmission areas rarely result in fever [19]. Therefore, elimination of malaria is highly unlikely if diagnostic strategies do not include asymptomatic patients, because they will remain a reservoir of parasites contributing to the spread of the disease from one malaria season to the next. Man¬agement and control of MiP and AiP are enhanced by the availability of local prevalence statistics, which is not adequately provided in Kwale County except the data collected in passive surveillance. It is in this background we carried out this study to estimate the prevalence of asymptomatic MiP and AiP and identify the associated factors among women attending their first ANC visit at the largest referral health facility in Kwale County.

e. What is the most common species of malaria parasites in Kenya? I suppose “P. falciparum”. Hence, it would interest to adjust the title of manuscript accordingly.
Has adjusted the title by including the “asymptomatic Plasmodium falciparum infection”

Concern 5Methods
a) Ethical statement should be presented in the main text.

i) Did the pregnant women receive IPTp at the 1st ANC if they were eligible?
Yes, they did, as per the government of Kenya Policy in prevention and control of Malaria in Pregnancy. .http://www.nmcp.or.ke/index.php/malaria-in-pregnancy

ii. Did the pregnant women with asymptomatic malaria receive curative treatment? What and how (uncomplicated and severe malaria)? If not, why? Same concerns regarding the anaemia, particularly severe cases?Following informed consent, the study participants were explained whose test positive for malaria and will have anemia will benefit by being treated as per the guidelines of malaria and anemia in pregnancy with no extra cost.

iii. Any written informed consent? Any ethical committee approval?

We sought written, informed consent from each participant before interviewing and finger pricking for malaria blood slides and Hemoglobin level analysis. Permission was granted to conduct the study by Kwale Department of Health and Msambweni Hospital Director. Ethical clearance was obtained from the Pwani University -Ethical Review Committee (ERC/MSc/021/2018).

Powered by Editorial Manager® and ProduXion Manager® from Aries Systems Corporation

b. Study population: give more detail on the strategy of participant's selection
Sampling procedures
Systematic random sampling method was used to select study participants. Our sampling interval was based on the daily entries in the mother-child health (MCH) register from September 2018 to February 2019. The sampling started by selecting a participant from the daily entries list at random using a table of random numbers and then every kth participant in the frame was selected. A selection interval (k) was determined by dividing the total daily entry listed in order to get the number of the participants required per day. If a randomly-selected participant was not eligible for an interview or refused to be part of the study, the next eligible participant on the list was selected. We sampled our study participants until we arrived at our desired sample size of 308.
Following informed consent, the study participants were explained whose test positive for malaria and anemia will benefit by being treated as per the guidelines of malaria and anemia in pregnancy with no extra cost.
c. Did you consider among the symptoms of malaria the history of fever the past 48 h before the visit?

Exclusion criteria: Pregnant women who had taken antimalarial drugs within the past two weeks were excluded. What about the women who had taken fever drug? There is a risk to consider women asymptomatic while they just took fever drug the day before the visit.Asymptomatic malaria was defined as the presence in the peripheral blood of asexual blood stage of Plasmodium, irrespective of species but has no symptoms of malaria per clinical assessment (i.e. temperature <37.50C, chills, rigor, nausea, vomiting, headache, anorexia, or joint/muscle pains) and has not taken antipyretics within 48 hours and antimalarials within 14 days.

Concern 6e. Sample size: The authors have considered a prevalence of MiP from a study in Burkina Faso (24%) while the malaria transmission is different to both countries.
True, we took the prevalence of MiP for Burkina Faso (24%). To our best of our knowledge by the time we were conceptualizing the protocol there was no study for asymptomatic MiP we could get hence we used the African study similar to ours to calculate the prevalence. Also, we thought the differences in malaria prevalence between Kenya and Burkina Faso are more likely related to malaria transmission intensity due to we have similar climate is characterized by 'long rains' (April–June) and 'short rains' (October–December) rainy seasons.

f. Data collection: give more details on socio-demographic characteristics, obstetric and clinical history.

Variables collected included;
Socio-demographic characteristics; mother's age, education level, marital status and occupation.
Obstetrics variables; gravidity, parity, trimesters, and gestational age in weeks.
Clinical history variables; history of fever in the last 48 hours and taken anti pyretic drugs, whether the client has taken antimalarials drugs, tendency of geophagy, whether patient is on iron supplements

How did you assess the LLIN use?
We depended on what the study participant reported which has been highlighted as a limitation but we used a trained nurse on the protocol to collect the data.

Please define the different trimesters of pregnancy (1st, 2nd, 3rd)?

First trimester was defined as; from week 1 to the end of week 12 while the second trimester is from week 13 to the end of week 26 and the third trimester is from week 27 to the end of the pregnancy
Give more details on how the gestational age was assessed?We used fundal height and last monthly period to estimate the gestational age

Powered by Editorial Manager® and ProduXion Manager® from Aries Systems Corporation

	Concern 7g. Quality control: Please precise if the 10% of slides chosen was for all sides or positive slides.An independent qualified parasitologist examined 10% of both positives and negatives slides which were randomly selected<br><br><br>Concern 8h. Statistical analysis: What procedure did you use for variable selection in the final model (multivariate model)?<br>Variables with p-value ≤ 0.20 were included in a logistic regression model using a backward stepwise elimination method to identify independently associated factors.<br><br><br>Concern 9<br>7) Results<br><br>a. Table 1: The proportion of pregnant women in the first trimester at the 1st ANC visit was 9.4% while the authors found that the proportion of pregnant women with gestational age < 16 wg was 15.9%. Why this discrepancy when the first trimester finished at 15 wg.<br><br>b. Table 2:<br>i. Why did you keep in the final multivariate model, the variable gestational age even if not significant in bivariate analysis?<br>ii. In the same way, why did you keep in the multivariable model “slept under bed net previous night” and “frequency of sleeping under bed net”. Both variables seems to be correlate.<br>iii. However, you drop out the variable “gravidity” which should be forced in the final model even if not significant because it’s a well-know factors strongly associated with MiP. Furthermore, I would like to suggest to the authors to make an sensitivity analysis by pooling primigravidae and secundigravidae in comparison to multigravidae.<br><br>c. Prevalence and factors associated with latent malaria:<br>i. Please define latent malaria?<br>ii. What is the prevalence of asymptomatic malaria among pregnant women in the 1st, 2nd and 3rd trimesters at the first ANC visit?<br>iii. What are the proportion of different species of parasites (P.f.; P.o; P.v; P.m)<br>iv. Please define POR at the first time it use in the text.<br><br>d. Prevalence and factors associated with anaemia:<br>i. Among the 3.6% of severe anaemia, how many<br><br><br><br><br>a)The difference is due to the definition of the trimesters that was used in this study. First trimester was defined as; from week 1 to the end of week 12 while the second trimester is from week 13 to the end of week 26 and the third trimester is from week 27 to the end of the pregnancy<br><br><br><br><br><br>b)Have reanalyzed the data (see table 2 and 3)

Powered by Editorial Manager® and ProduXion Manager® from Aries Systems Corporation

c)Similar to the answer b, have re- analyzed the data (Table 2) and pooled together the primigravidae and second gravidae in comparison with multigravidae but still was not significant

we have used I have replaced latent malaria with asymptomatic malaria

Gestational trimesters with plasmodium infections, the first trimester were 2/29 (6.9%), second trimester 24/173 (13.9%) and third trimester were 14/106 (13.2%).

Plasmodium falciparum were 35 (87.5%), Plasmodium malarie 3 (7.5%) and Plasmodium ovale 2 (5.0%).
I has been defined as Prevalence Odds Ratio

Those who had severe anemia 4/7 (57.1%) had malaria, moderate anemia 15/95 (15.8%) and mild anemia was 14/90 (15.6%).

Discussion
a. Regarding the factors associated to MiP:
i. The authors should also discuss what happens among women in the first trimester of pregnancy. We can observe that women are more at risk of infection than those in 2nd and 3rd trimester (19.7% vs. 12.7% and 10.4%, respectively).
The current study reported a higher proportion of Plasmodium infections in pregnant women who were both in second and third trimesters and less proportion to the first trimester. Studies in Nigeria have reported high malaria prevalence in pregnant women who were in their second trimesters [27-29]. A study in Mali reported pregnant women in their first trimester were two times more likely to get malaria compared to the third trimester [30]. In contrast, our study reported less proportion of pregnant women in their first trimester and was not associated with malaria. This was probably due to the small number of pregnant women among this category. With an increase in the number of pregnant women in their first trimester, there is the possibility that there could be changes from the present results.

ii. The only factor associated with MiP is young age (< 20 y). The authors should consider to check an interaction between age and gravidity as both are correlated. Hence, this could be explained by that young pregnant women are mostly primigravidae? This deserves a couple of sentence in the discussion.
There was no Interaction
In this study, the highest proportional of Plasmodium infections was observed among the primigravidae (19.7%): followed by secundigravidae (12.7.7%) and multigravidae (10.4 %) with parasitaemia declining with increasing gravidity. These results are consistent with previous reports which found plasmodium infections are more common in primigravidae women compared to multigravidae women [7, 28, 31]. The reason for the present result of gravidae-associated predisposition to P. falciparum infections may be due to the fact that adults who live in malaria-endemic regions generally have some

Powered by Editorial Manager® and ProduXion Manager® from Aries Systems Corporation

	acquired immunity to malaria infection due to immunoglobulin production stimulated by previous malaria infection. This acquired immunity diminishes significantly in pregnancy particularly in primigravidae. It has also been suggested by various authors that the early onset of antibody response in multigravidae and the delayed antibody production in primigravidae may be responsible for the gravidity-dependent and differential prevalence of falciparum malaria among pregnant women [19, 32].<br><br><br>b. Regarding the factors associated to AiP: First and 3rd trimester are both associated with AiP. This could be also explained by the haemoglobin level variation due to physiopathology of the pregnancy. This should be included in the discussion<br>We found that anemia is more common among women in their third trimester than among women in their first trimester, similar to findings reported in other studies [39, 40]. Hemoglobin decreases until the end of the third-trimester. This might be due the fact that increase in trimester may cause reduction in maternal iron reserves. Anemia is a function of plasma volume and red cell mass; both of which increase during pregnancy; but the increase in plasma volume is proportionately greater than the increase in red cell mass [41]. Also, anemia in the third trimester may be more likely due to higher nutrient demands of the fetus later in pregnancy<br><br>c. Study limitations: The authors have stated several limitations for the study. It is a good point. However, they have to explain how they have controlled this bias to ensure the validity of the study.<br><br>We collected data from September 2018 to February 2019, a period during which there is low malaria transmission. This could have resulted in the underestimation of the overall prevalence of asymptomatic MiP in our study area. A continuous monitoring throughout the year of MiP incidences will account for seasonality burden [21].<br>In addition, the study was hospital-based, excluding pregnant women who did not seek ANC services. While this may limit the generalizability of findings to the community, few women fail to seek antenatal care in our study area. Determination of factors associated with asymptomatic MiP and AiP in hospital based studies provides a proxy indicator of predictors in the community of that particular facility when community based surveys are not feasible.<br>Lastly, this study did not explore other factors that may contribute to anemia, including nutritional factors, soil-transmitted helminthes infection, and hereditary conditions such as sickle cell disease thus limiting our ability to assess the contribution of other causes of anemia during pregnancy. However, diagnosis of anemia was based on laboratory analysis and did not depend on clinical assessment as reported by other researchers.<br><br><br>Concern 109) Conclusion: The authors should revise their conclusion in order to highlight the originality of the study.<br><br>Asymptomatic Plasmodium infections and anemia are common in women attending their first ANC visit at Msambweni County Referral Hospital in Kwale County. Most of the Plasmodium infections in this area are caused by P. falciparum. Asymptomatic MiP was associated with younger maternal age (≤20 years). Anemia in pregnancy was associated with Plasmodium infections, women who reported to have geophagy tendency and those who were their third trimester. In the study area, we recommend pregnant women should not delay their first ANC attendance, for less than 10% attended in their first trimester. All women of childbearing age should be included in measures to control Plasmodium infection and anemia by the National Malaria Control Program, reproductive health department and other non – state actors should. Also, the reproductive health department should carry out health promotion and education on late adolescent and school going pregnancy for delay of sexual debut.
**Additional Information:**	
**Question**	**Response**
**Financial Disclosure**<br><br>Enter a financial disclosure statement that	The author(s) received no specific funding for this work.

describes the sources of funding for the work included in this submission. Review the submission guidelines for detailed requirements. View published research articles from *PLOS ONE* for specific examples.<br><br>This statement is required for submission and **will appear in the published article** if the submission is accepted. Please make sure it is accurate.<br><br>**Unfunded studies**<br>Enter: *The author(s) received no specific funding for this work.*<br><br>**Funded studies**<br>Enter a statement with the following details:<br>• Initials of the authors who received each award<br>• Grant numbers awarded to each author<br>• The full name of each funder<br>• URL of each funder website<br>• Did the sponsors or funders play any role in the study design, data collection and analysis, decision to publish, or preparation of the manuscript?<br>• **NO** - Include this sentence at the end of your statement: *The funders had no role in study design, data collection and analysis, decision to publish, or preparation of the manuscript.*<br>• **YES** - Specify the role(s) played.<br><br>* typeset	
**Competing Interests**<br><br>Use the instructions below to enter a competing interest statement for this submission. On behalf of all authors, disclose any competing interests that could be perceived to bias this work—acknowledging all financial support and any other relevant financial or non-financial competing interests.<br><br>This statement **will appear in the published article** if the submission is accepted. Please make sure it is accurate. View published research articles from *PLOS ONE* for specific examples.	The authors have declared that no competing interests exist.

**NO authors have competing interests**<br><br>Enter: *The authors have declared that no competing interests exist.*<br><br>**Authors with competing interests**<br><br>Enter competing interest details beginning with this statement:<br><br>*I have read the journal's policy and the authors of this manuscript have the following competing interests: [insert competing interests here]*<br><br>* typeset	
**Ethics Statement**<br><br>Enter an ethics statement for this submission. This statement is required if the study involved:<br><br>• Human participants<br>• Human specimens or tissue<br>• Vertebrate animals or cephalopods<br>• Vertebrate embryos or tissues<br>• Field research<br><br>Write "N/A" if the submission does not require an ethics statement.<br><br>General guidance is provided below. Consult the submission guidelines for detailed instructions. **Make sure that all information entered here is included in the Methods section of the manuscript.**	We sought written, informed consent from each participant before interviewing and finger pricking for malaria blood slides and Heamoglobin level analysis. Permission was granted to conduct the study by Kwale Department of Health and Msambweni Hospital Director. Ethical clearance was obtained from the Pwani University -Ethical Review Committee (ERC/MSc/021/2018).

Powered by Editorial Manager® and ProduXion Manager® from Aries Systems Corporation

**Format for specific study types**<br><br>**Human Subject Research (involving human participants and/or tissue)**<br>• Give the name of the institutional review board or ethics committee that approved the study<br>• Include the approval number and/or a statement indicating approval of this research<br>• Indicate the form of consent obtained (written/oral) or the reason that consent was not obtained (e.g. the data were analyzed anonymously)<br><br>**Animal Research (involving vertebrate animals, embryos or tissues)**<br>• Provide the name of the Institutional Animal Care and Use Committee (IACUC) or other relevant ethics board that reviewed the study protocol, and indicate whether they approved this research or granted a formal waiver of ethical approval<br>• Include an approval number if one was obtained<br>• If the study involved *non-human primates*, add *additional details* about animal welfare and steps taken to ameliorate suffering<br>• If anesthesia, euthanasia, or any kind of animal sacrifice is part of the study, include briefly which substances and/or methods were applied<br><br>**Field Research**<br><br>Include the following details if this study involves the collection of plant, animal, or other materials from a natural setting:<br>• Field permit number<br>• Name of the institution or relevant body that granted permission	
**Data Availability**<br><br>Authors are required to make all data underlying the findings described fully available, without restriction, and from the time of publication. PLOS allows rare exceptions to address legal and ethical concerns. See the PLOS Data Policy and FAQ for detailed information.	Yes - all data are fully available without restriction

A Data Availability Statement describing where the data can be found is required at submission. Your answers to this question constitute the Data Availability Statement and **will be published in the article**, if accepted.<br><br>**Important:** Stating 'data available on request from the author' is not sufficient. If your data are only available upon request, select 'No' for the first question and explain your exceptional situation in the text box.<br><br>Do the authors confirm that all data underlying the findings described in their manuscript are fully available without restriction?	
**Describe where the data may be found in full sentences. If you are copying our sample text, replace any instances of XXX with the appropriate details.**<br><br>• If the data are **held or will be held in a public repository**, include URLs, accession numbers or DOIs. If this information will only be available after acceptance, indicate this by ticking the box below. For example: *All XXX files are available from the XXX database (accession number(s) XXX, XXX.*).<br>• If the data are all contained **within the manuscript and/or Supporting Information files**, enter the following: *All relevant data are within the manuscript and its Supporting Information files.*<br>• If neither of these applies but you are able to provide **details of access elsewhere**, with or without limitations, please do so. For example:<br><br>*Data cannot be shared publicly because of [XXX]. Data are available from the XXX Institutional Data Access / Ethics Committee (contact via XXX) for researchers who meet the criteria for access to confidential data.*<br><br>*The data underlying the results presented in the study are available from (include the name of the third party*	The data set has been uploaded

and contact information or URL).<br>• This text is appropriate if the data are owned by a third party and authors do not have permission to share the data.<br><br>* typeset	
Additional data availability information:	

Manuscript

Click here to access/download;Manuscript;Manuscript_01_07_2020.doc

Title: Prevalence and risk factors associated with asymptomatic Plasmodium *falciparum* infection and anemia among pregnant women at the first antenatal care visit: A hospital based cross-sectional study in Kwale County, Kenya.

Authors; Gibson Waweru[1, 2,] Jimmy Kihara[3], Elvis Oyugi[4], Victor Omballa[5], Hajara Busaidy[2], Victor Jeza[1]

Affiliations;

[1]*Technical University of Mombasa, Kenya*

[2]*Departrtment of Health, Kwale County, Kenya*

[3]*Kenya Medical Research Institute, Kenya*

[4]*Kenya Field Epidemiology and Laboratory Training Program, Ministry of Health, Kenya*

[5]*Center for Global Health Research - Kenya Medical Research Institute, Nairobi, Kenya*

Corresponding author
Mr. Gibson Nyamu, Msc
Head of the Kwale County Malaria Reference Laboratory,
Kwale County.
Tel: +254720633603
Fax: NA
Email: wawerugibson2015@gmail.com

Abstract

Background: Prevalence of both asymptomatic and symptomatic malaria in pregnancy (MiP) in Kenya ranges from 9% to 18%. We estimated the prevalence and factors associated with MiP and anemia in pregnancy (AiP) among asymptomatic women attending antenatal care (ANC) visits.

Methods: We performed a cross-sectional study among asymptomatic pregnant women attending ANC at Msambweni Hospital, between September 2018 and February 2019. Data was collected and analyzed in Epi Info 7. Descriptive statistics were calculated and we compared MiP and AiP in asymptomatic cases to those without either condition. Adjusted prevalence Odds ratios (aPOR) and 95% confidence intervals (CI) were calculated to identify factors associated with asymptomatic MiP and AiP.

Results: We interviewed 308 study participants; their mean age was 26.6 years (± 5.8 years), mean gestational age was 21.8 weeks (± 6.0 weeks), 173 (56.2%) were in the second trimester of pregnancy, 12.9 % (40/308) had MiP and 62.7 % had AiP. Women who were aged ≤ 20 years had three times likelihood of developing MiP (aPOR= 3.1 Cl: 1.3 - 7.35) compared to those aged >20 years old. The likelihood of AiP was higher among women with gestational age ≥ 16 weeks (aPOR=3.9, CI: 1.96 – 7.75), those with parasitemia (aPOR= 3.3, 95% CI: 1.31-8.18), those in third trimester of pregnancy (aPOR= 2.6, 95% CI:1.40 - 4.96) and those who reported eating soil as a craving during pregnancy (aPOR=1.9, 95%CI:1.15 - 3.29)..

Conclusions: Majority of the women had asymptomatic AiP and MiP was observed in one tenth of all study participants. Asymptomatic MiP was associated with younger age while AiP was associated with gestational age parasitemia, and soil consumption as a craving during pregnancy.

Key words: Anemia, Asymptomatic malaria, Pregnant, Kenya.

Introduction

The commonest plasmodium species that is known to cause malaria in pregnancy (MiP) in Africa is *Plasmodium falciparum* which can lead to anemia in pregnancy (AiP) [1]. The World Health Organization (WHO) in 2019, reported 11 million pregnant women were infected with plasmodium infection in Sub – Saharan Africa, resulting in 872 000 low birth weights [2]. In 2019, the Ministry of Health (MOH), Kenya, estimated MiP to be 89,324/1,42,896 (6.3%) among women attending their first antenatal care (ANC) visit [3]. In Kwale County, Kenya, MiP remains a public health concern with a total of 2,316 in 2019 [3]. Anemia in Pregnancy is a well-known risk factor for maternal death, stillbirths, low birth weights and infant prematurity [4-6]. Previous studies have reported associations between malaria with AiP [7, 8] while consumption of soil (geophagy) has been associated with AiP among African women [9, 10]. Besides plasmodium infections, other known causes of AiP include nutritional deficiencies, infectious diseases like HIV, parasitic infections like hookworm infestation, and hemoglobinopathies [4, 10].

Malaria in Pregnancy constitutes a major risk to the mother, fetus, and neonates including stillbirths, spontaneous abortion, premature delivery, maternal anemia, and low birth weight [2, 11]. Due to physiological and immunological changes, pregnant women have been found to have a higher risk of malaria compared to non-pregnant women living in areas of similar malaria endemicity [12]. Factors associated with MiP are less parity, young age, low maternal education level, early gestational age, young maternal age, fewer previous pregnancies, non-ownership or infrequent use of bed nets and maternal unemployment [7, 11, 13-15].

Focused antenatal care is a package given to pregnant women which entails giving prophylaxis and treatment for anemia and malaria among other services, where the AiP is prevented by providing nutritional counseling including iron supplements, and treating cases of AiP [16]. Interventions aimed at prevention and control of MiP adopted by the MOH– Kenya are; Intermittent Preventive Treatment (IPTp) of MiP, with Sulfadoxine Pyrimethamine (SP) given after 12 weeks gestational period done four weeks apart until the pregnant woman delivers. In areas with high malaria transmission such as Western, Nyanza, and Coast regions, Long-lasting Insecticidal Nets (LLINs) are provided at the ANC during the first contact [17]. Kenya Malaria Indicator Survey (KMIS) 2015 reported two folds increase in the prevalence of malaria in the coastal region compared to KMIS 2010 [18]. In high-transmission regions like coastal regions in Kenya, where levels of acquired immunity tend to be high

P. *falciparum* infection is usually asymptomatic in pregnancy. However, parasites may be present in the placenta and contribute to AiP even in the absence of documented peripheral parasitaemia [19]. Therefore, elimination of malaria is highly unlikely if diagnostic strategies do not include asymptomatic patients, because they will remain a reservoir of parasites contributing to the spread of the disease from one malaria season to the next. Management and control of MiP and AiP are enhanced by the availability of local prevalence statistics, which is not adequately provided in Kwale County except the data collected in passive surveillance according to records in the County. It is from this background that we carried out this study to estimate the prevalence of asymptomatic MiP and AiP and identify the associated factors among women attending their first ANC visit at the largest referral health facility in Kwale County, Kenya.

Methods

Study location

Kwale County is one of six counties in the coastal region of Kenya covering an area of 8,270.3 km^2 with a population of 866,820 people [20]. The inhabitants are predominantly Muslim, from the Mijikenda tribe, and practice subsistence farming and smallholder animal husbandry. The weather is hot and humid with two rainy seasons: long rains from April to June and short rains from October through November. The incidence of malaria increases during the long rainy seasons [21].

Msambweni County Referral Hospital (MCRH) is the main referral health facility in Kwale County (Figure 1). The hospital has 155 inpatient beds and189 healthcare workers. Four nurses work at the Mother-Child Health department, who attend on average 125 mothers each month [3].

Study design

We conducted across-sectional study to determine the prevalence of asymptomatic MiP and AiP.

Study population

Pregnant women attending their first ANC visit at MCRH between September 2018 and February 2019.

Inclusion criteria and exclusion criteria

Pregnant women seeking their first ANC were included in the study, especially those with no symptoms of malaria as per clinical assessment (i.e. no fever (temperature >37.5°C), chills, rigor, nausea, vomiting, headache, anorexia, or joint/muscle pains).

We excluded pregnant women who had taken anti-malarial drugs within the past two weeks, antipyretics in case they had fever and those receiving micronutrient.

Definitions of terms

- Asymptomatic malaria was defined as the presence in the peripheral blood of asexual blood stage of *Plasmodium*, irrespective of species but has no symptoms of malaria per clinical assessment and has not taken antipyretics within 48 hours and anti-malarials within 14 days.
- A young age was defined as age ≤ 20 years
- Anemia was defined as a hemoglobin < 11 g/dl while mild anemia (10 - 10.9g/dl), moderate anemia (7 - 9.9g/dl) and severe anemia (<7g/dl)
- First **trimester** was defined as; from **week** 1 to the end of **week** 12 while the second **trimester** as from **week** 13 to the end of **week** 26 and the third **trimester** as from **week** 27 to the end of the pregnancy

Sample size determination

Cochran's formula [22] was used to calculate the sample size required to estimate the prevalence of asymptomatic malaria in pregnant women attending their first ANC visit.

Assumptions made were:

- Two-sided significance level defined at 5%
- Power or percentage of detection defined at 80%
- The prevalence of asymptomatic MiP to be 24% [7].
- We adjusted by 10% to cater for those who refused to be enrolled. We calculated the desired sample size as 308 participants.

Sampling procedures

Systematic random sampling method was used to select study participants. Our sampling interval was based on the daily entries in the mother-child health (MCH) register from September 2018

to February 2019. The sampling started by selecting a participant from the daily entries list at random using a table of random numbers and then every k^{th} participant in the frame was selected. A selection interval *(k)* was determined by dividing the total daily entry listed in order to get the number of the participants required per day. If a randomly-selected participant was not eligible for an interview or refused to be part of the study, the next eligible participant on the list was selected. We sampled our study participants until we arrived at our desired sample size of 308.

Following signing of informed consent, the study participants were explained that those who tested positive for malaria and have anemia would benefit by being treated as per the guidelines of malaria and anemia in pregnancy at no extra cost.

Data collection

In-person interviews were conducted using a pre-tested structured questionnaire. The questionnaire was developed in English, and translated to Swahili for non-English speaking respondents.

Variables collected were;

- **Socio-demographic characteristics;** mother's age, education level, marital status and occupation
- **Obstetrics variables;** gravidity, parity, trimesters, and gestational age in weeks.
- **Clinical history variables;** history of fever in the last 48 hours and taken anti pyretic drugs, whether the client has taken antimalarial drugs within the last 2 weeks, tendency of geophagy, and whether or not on iron supplements.

Laboratory methods

Hemoglobin testing

The index finger was cleaned using 70% isopropyl alcohol and pricked using a sterile lancet by well-trained laboratory technicians. The first drop was wiped away using sterile cotton wool, and then the finger was gently squeezed to obtain approximately 30µl drop of blood onto a micro-cuvette and subsequently into a portable heme-analyzer (Hem cue Hb 301, Hemo Cue AB 16, Sweden). To determine anemia status, Hb measurement was obtained within 45 seconds and reported in grams per deciliters (gm/dl).

Malaria testing

Thick and thin blood films were prepared. Absolute methanol was used to fix thin films and Giemsa stain (3%) staining for 30 minutes. The slides were then rinsed with distilled water and air-dried at room temperature. Slides were then viewed under the microscope using 100x objectives on immersion oil. No Parasite Found (NPF) was reported after 100 fields were examined and no malaria parasites observed.

Thick films were examined to determine the presence of asexual malaria parasites, quantification of malaria parasites was done by enumerating asexual malaria parasites against 200 White blood cells (WBC). Then, Parasite densities (parasite per microliter of blood) were determined at after multiplying with an assumed WBC count of 8.0_109/l, with the product of numbers of malaria parasites divided by 200 WBC [23]. Also, the speciation of the *Plasmodium* parasites was done.

Quality Control

Quality of Giemsa stain was maintained by testing known positives slides. An independent qualified parasitologist examined 10% of both positives and negatives slides which were randomly selected and any variation were corrected before final reporting. Quality control of our hematology analyzer (HemocueHb 301, Hemo Cue AB 16, Sweden) was performed per as the

manufacturer instructions, by analyzing dried samples with known Hb levels before testing participant samples.

Data management and analysis

Data were entered, cleaned, and rechecked using MS Excel 2013. Data were analyzed using Epi Info 7. The following descriptive statistics were calculated: frequencies and proportions for categorical variables, and measures of central tendency (mean, median, and mode) and dispersion (range, interquartile range, and standard deviation) for continuous variables.

We tested the relationship between a variety of predictor variables, including socio-demographic factors and clinical history, and malaria status as the outcome variable, comparing participants who tested malaria positive with those who tested negative. We also compared participants who had anemia to those who did not. Both crude prevalence ratio (cPOR) and adjusted prevalence odds ratio (aPOR), and their 95% confidence intervals were calculated. Variables with $p < 0.05$ were considered statistically significant. Variables with p-values ≤ 0.20 were included in a logistic regression model using a backward stepwise elimination method to identify independently associated factors.

Ethics approval and consent to participate

We sought written, informed consent from each participant before interviewing and finger pricking for malaria blood slides and Hemoglobin level analysis. Permission was granted to conduct the study by Kwale Department of Health and Msambweni Hospital Director. Ethical clearance was obtained from the Pwani University – Ethical Review Committee (ERC/MSc/021/2018).

Results

Socio-demographic characteristics of respondents

A total of 308 respondents were interviewed. Their mean age was 26.6 years (± 5.8 years) , 267 (86.9%) were married, 83 (26.9 %) had > 8 years of formal schooling, and among them, 29 (9.4%) were formally employed. The mean gestational age was 21.8 weeks (± 6.0 weeks), 29 (9.4%) were in first trimester, 173 956.2%) were in second trimester and 106 (34.4%) were in third trimester of pregnancy.. Those who were primi-gravidae were 66 (21.4%), second-gravidae were 79 (25.7%) and multi-gravidae were 163 (52.9%) (Table1).

Among the participants, 248 (80.5%) owned bed nets (treated or untreated). Of these, 109 (44.3%) had used a bed net for less than 6 months, 34 (13.8%) had used a bed net for 6 - 12 months, and 103 (41.8%) had used a bed net for > 12 months. In terms of bed net usage in the current pregnancy, 231 (93.2%) reported having slept under a bed net the previous night while 205 (82.7%) reported always sleeping under a bed net, and 43(17.3%) reported sometimes sleeping under a bed net (Table 1).

Prevalence and factors associated with asymptomatic malaria

Malaria positivity among the 308 study participants was 12.9% (40/308) and the geometric mean parasite count was 3738 parasites per microliter of blood; 35 (87.5%) tested positive for *Plasmodium falciparum* , 3 (7.5%) *Plasmodium malariae* and 2 (5.0%) *Plasmodium ovale* . In regard to gestational trimesters with plasmodium infections, those in the first trimester were 2/29 (6.9%), second trimester 24/173 (13.9%) and those in the third trimester were 14/106 (13.2%).

The odds of asymptomatic MiP was higher in women who were aged ≤ 20 years (cPOR= 3.5, 95% CI= 1.65 – 7.23), women who did not own bed nets (cPOR= 2.3, 95% CI 1.08 – 4.69) and

women who owned bed nets but did not sleep under a bed net the night before the interview (cPOR= 2.4, 95% CI 1.14 – 5.03). After logistic regression analysis, asymptomatic MiP was independently associated with being age ≤ 20 years (aPOR= 4.5 (1.71– 12.01) compared with those aged >20 years (Table 2).

Prevalence and factors associated with anemia

Anemia was reported in 193 (62.7%) participants, and the mean Hb was 9.6 mg/dl (± 1.3mg/dl); 96 (49.7%) had moderate anemia, 90 (46.6%) had mild anemia and seven (3.6%) had severe anemia. Among those with severe anemia four had malaria, moderate anemia 15/95 (15.8%) and mild anemia was 14/90 (15.6%). Geophagy was reported by 117 (38.6%) participants. Those with a gestational age of ≥ 16 weeks had greater odds of AiP, cPOR= 3.2 (1.72 – 6.07) compared to those with gestational age <16 weeks. Those who reported eating soil had greater odds of AiP, cPOR= 2.1 (1.27 – 3.45) compared with those who did not report eating soil. Following logistic regression analysis, AiP was independently associated with gestational age ≥ 16 weeks of gestational period had (aPOR; 3.3, 95% CI: 1.72– 6.41), and those who reported eating soil (aPOR 2.0, 95% CI: 1.21– 3.41) Table 3.

Pregnant women with malaria parasitemia were three times more likely to have anemia compared to those without malaria parasitemia (χ^2=2.79, P-value=0.005, (aPOR; 3.5, 95% CI: 1.21– 8.60) (Table 4).

Discussion

Numerous studies have shown that anemia and malaria contribute to morbidity and mortality among pregnant women. In this study, we found that one in eight women had asymptomatic MiP and more than half of the women had AiP. Asymptomatic MiP was associated with young age (≤ 20 years). Anemia prevalence was also associated with pregnant women who reported eating soil, were in their first and third trimesters of pregnancy, and had *P. falciparum* infections.

The prevalence of asymptomatic MiP in our study population was 12.9%, which is similar to the prevalence among pregnant women in Ethiopia (9.1%) [24] but lower than the prevalence found in Burkina Faso (24%) among pregnant women [7]. In this study, we included only pregnant women seeking ANC services for the first time in their current pregnancy and majority of study participants (82.7%) reported to always using bednet, whereas the study in Burkina Faso included pregnant women seeking ANC services at any point in their pregnancies. These may be factors that lead in the difference between these two reported prevalence.

In this study, we found that the odds of MiP among young women (≤ 20 years of age) were greater than the odds of MiP among older women. This may reflect continuing development of malarial immunity [14, 25, 26]. Contrast to our findings, a study in Gabon showed that there is no significant difference was found between younger and older women [27].

The current study reports higher proportion of *P. falciparum* infections in pregnant women who were both in second and third trimesters and less proportion in the first trimester. Studies in Nigeria have also reported high malaria prevalence in pregnant women who were in their second trimesters [28-30]. Another study in Mali reported pregnant women in their first trimester were two times more likely to get malaria compared to the third trimester [31]. In contrast, our study reported less proportion of pregnant women in their first trimester was not associated with

malaria. This was probably due to the small number of pregnant women among this group. With an increase in the number of pregnant women in their first trimester, there is the possibility that there could be changes from the present results.

This study is showing highest proportional of Plasmodium infections was observed among the primi-gravidae (19.7%) followed by secundi-gravidae (12.7.7%) and multi-gravidae (10.4 %) with parasitaemia declining with increasing gravidity. These results are consistent with previous studies which found *Plasmodium* infections are more common in primi-gravidae women compared to multi-gravidae women [7, 29, 32]. The reason for the present result of gravidae-associated predisposition to *P. falciparum* infections may be due to the fact that adults who live in malaria-endemic regions generally have some acquired immunity to malaria infection. This acquired immunity diminishes significantly in pregnancy particularly in primi-gravidae. It has also been suggested by various authors that the early onset of antibody response in multi-gravidae and the delayed antibody production in primi-gravidae may be responsible for the gravidity-dependent and differential prevalence of *falciparum* malaria among pregnant women [19, 33].

Intervention measures for first visit pregnant women at antenatal clinic for malaria in pregnancy are; IPTp and provision of LLINs among others [17]. Although the role of IPTp is known to reduce maternal malaria episodes and improve pregnancy outcomes [34], the current study did not include pregnant women who had taken IPTp hence we could not ascertain the role played by IPTp. Our study evaluated through interviewing study participants on bed net ownership and usage. Majority of our respondents owned, slept under a bed net and almost all participants reported sleeping under a bed net the previous night. The KMIS, 2015, reported similar high rates of bed net ownership and use in pregnant women living in malaria-endemic zones, with

83.7% of pregnant women reporting sleeping under a bed net the night before they were interviewed [18]. Bed net ownership and usage have been reported in several studies to be protective against malaria infections [26, 35]. Our observed high rate of bed net ownership may be the result of a Kenyan national malaria policy which stipulates that pregnant women should routinely be issued with bed nets at their first ANC visits. In addition, Kenya's Malaria Control Program recently conducted a mass net distribution campaign, and all households were issued bed nets in Kwale County. Bed nets ownership have shown to be protective for malaria [36], in this study those pregnant women reported had no bed nets had 90 % higher odds of asymptomatic MiP compared to those who owned bed nets, though was not statistically significant.

The study reported more than half of the respondents (60%) had AiP. The etiology of anemia is variable and potentially multi-factorial, and thus several underlying morbid and co-morbid conditions may contribute to the prevalence of anemia. Similar to our findings, one study conducted in the Pumwani maternity hospital in Nairobi, Kenya reported an AiP prevalence of 57% [37], and another study in southern Ethiopia reported an AiP prevalence of 60% [34]. Lower prevalence of AiP have also been reported in southwest Ethiopia (23.5%) and northwest Ethiopia (16.5%) [38, 39].

The present study found that a significant number of women with asymptomatic MiP had AiP. Malaria in pregnancy is known to cause AiP, this association has also been reported in other studies in sub-Saharan Africa [7, 8].

We also found high reported rates of eating soil (geophagy), consistent with other studies that have found high rates of geophagy among pregnant African women as well as associations between geophagy and anemia [9, 10].

Our results indicate that, anemia is more common among women in their third trimester than women in their first trimester, similar to findings reported in other studies [40, 41]. Hemoglobin decreases through to the end of the third-trimester. Anemia is a function of plasma volume and red cell mass; both of which increase during pregnancy; but the increase in plasma volume is proportionately greater than the increase in red cell mass [42]. This might be due the fact that increase in trimester may cause reduction in maternal iron reserves. Also, anemia in the third trimester may be more likely due to higher nutrient demands of the fetus later in pregnancy.

Our study had several limitations. We collected data from September 2018 to February 2019, a period during which there is low malaria transmission. This could have resulted in the under estimation of the overall prevalence of asymptomatic MiP in our study area. A continuous monitoring throughout the year of MiP incidences will account for seasonality burden [21].

In addition, the study was hospital-based, excluding pregnant women who did not seek ANC services. While this may limit the generalizability of findings to the community, few women fail to seek antenatal care in our study area. Determination of factors associated with asymptomatic MiP and AiP in hospital-based studies provides a proxy indicator of predictors in the community of that particular facility when community-based surveys are not feasible.

Lastly, this study did not explore other factors that may contribute to anemia, including nutritional factors, soil-transmitted helminthes infection, and hereditary conditions such as sickle cell disease thus limiting our ability to assess the contribution of other causes of anemia during pregnancy. However, diagnosis of anemia was based on laboratory analysis and did not depend on clinical assessment as reported by other researchers.

Conclusion

Asymptomatic *Plasmodium* infections and anemia are common in women attending their first ANC visit at Msambweni County Referral Hospital in Kwale County. Most of the *Plasmodium* infections in this area are caused by *P. falciparum*. We did not observe a clear gravidity pattern of asymptomatic MiP although was associated with younger maternal age (≤20 years). This may be possible although unlikely that selection bias may have influenced these findings. Anemia in pregnancy was associated with *Plasmodium* infections, women who reported to have geophagy tendency and those who were their third trimester. In the study area, we recommend pregnant women should not delay their first ANC attendance, for less than 10% attended in their first trimester. All women of childbearing age should be included in measures to control Plasmodium infection and anemia by the National Malaria Control Program, reproductive health department and other non–state actors. Also, the reproductive health department should carry out health promotion and education on late adolescent and school going pregnancy for delay of sexual debut.

Acknowledgments

We would like to thank the Technical University of Mombasa, Kenya, for their support during the study. Special thanks go to study participants and to the Kwale County Department of Health authorities for their collaboration. We also thank the many Vector Borne Disease Control Unit (VBDCU) Laboratory officers, including Said Lipi, Joyce Bandika, Peter Siema, Charles Ng'ang'a, and Elton Mzungu for their dedication and meticulous microscopy. We extend a special thank you to nurse Wendy Losier, for her assistance with the consenting process and data collection. We also thank Dr. Robert Perry, Dr. Elizabeth Wanja, and Dr. Shama Cash-Goldwasser for their valuable insight and comments on the manuscript.

Tables and figures

Figure 1: Map of the study area (Msambweni County Referral Hospital-MCRH)

Table 1: Socio demographic characteristics of women attending first antenatal care at Msambweni Hospital, Kwale County, Kenya.

Table 2: Factors associated asymptomatic malaria parasitaemia in Pregnant Women, Kwale County, Kenya.

Table 3: Factors associated with Anemia in Pregnant Women, Kwale County, Kenya.

Table 4: Chi-square analysis of proportions with and without malaria parasitemia and anemia.

References

1. Alaku, I., A. Abdullahi, and H. Kana, *Epidemiology of Malaria Parasites Infection among pregnant women in some part of Nasarawa State, Nigeria*. Developing Country Studies, 2015. **5**(2).
2. WHO. *WORLD MALARIA REPORT*
2019 14 th June 2020 [cited 2020 14th June]; Available from: https://www.who.int/malaria/publications/world-malaria-report-2019/en/.
3. DHIS2, *District Health Information Systems*. 2019.
4. McClure, E.M., et al., *The association of parasitic infections in pregnancy and maternal and fetal anemia: a cohort study in coastal Kenya*. PLoS neglected tropical diseases, 2014. **8**(2).
5. Helmy, M.E., N.I. Elkhouly, and R.A. Ghalab, *Maternal anemia with pregnancy and its adverse effects*. Menoufia Medical Journal, 2018. **31**(1): p. 7.
6. Uneke, C., D. Duhlinska, and E. Igbinedion, *Prevalence and public-health significance of HIV infection and anaemia among pregnant women attending antenatal clinics in south-eastern Nigeria*. Journal of health, population, and nutrition, 2007. **25**(3): p. 328.
7. Douamba, Z., et al., *Asymptomatic malaria correlates with anaemia in pregnant women at Ouagadougou, Burkina Faso*. BioMed Research International, 2012. **2012**.
8. Isah, A.Y., M.A. Amanabo, and B.A. Ekele, *Prevalence of malaria parasitemia amongst asymptomatic pregnant women attending a Nigerian teaching hospital*. Annals of African medicine, 2011. **10**(2).
9. Mathee, A., et al., *A cross-sectional analytical study of geophagia practices and blood metal concentrations in pregnant women in Johannesburg, South Africa*. South African Medical Journal, 2014. **104**(8): p. 568-573.
10. Stephen, G., et al., *Anaemia in pregnancy: prevalence, risk factors, and adverse perinatal outcomes in Northern Tanzania*. Anemia, 2018. **2018**.
11. Gajida, A., Z. Iliyasu, and A. Zoakah, *Malaria among antenatal clients attending primary health care facilities in Kano state, Nigeria*. Annals of African medicine, 2010. **9**(3).
12. Tarning, J., et al., *Population pharmacokinetics of dihydroartemisinin and piperaquine in pregnant and nonpregnant women with uncomplicated malaria*. Antimicrobial agents and chemotherapy, 2012. **56**(4): p. 1997-2007.
13. Olubukola, A., A. Odunayo, and A. Adesina, *Anemia in pregnancy at two levels of health care in Ibadan, south west Nigeria*. Annals of African medicine, 2011. **10**(4).
14. Agomo, C.O. and W.A. Oyibo, *Factors associated with risk of malaria infection among pregnant women in Lagos, Nigeria*. Infectious diseases of poverty, 2013. **2**(1): p. 19.
15. Nega, D., et al., *Anemia associated with asymptomatic malaria among pregnant women in the rural surroundings of Arba Minch Town, South Ethiopia*. BMC research notes, 2015. **8**(1): p. 110.
16. MOH. *Focused Antenatal Care,*. 2015 11th [cited 2020 June]; Available from: https://www.researchgate.net/publication/241510221.
17. MOH. *Malaria in Pregnancy*. 2020 June [cited 2020 14th]; Available from: http://www.nmcp.or.ke/index.php/malaria-in-pregnancy.
18. MOH. *Kenya Malaria Indicator Survey*. 2015 14 th June 2020 [cited 2020 14th]; Available from: https://dhsprogram.com/pubs/pdf/MIS22/MIS22.pdf.

19. Desai, M., et al., *Epidemiology and burden of malaria in pregnancy*. The Lancet infectious diseases, 2007. **7**(2): p. 93-104.
20. KNBS, *Kenya Population and Housing Census Volume I : Population By County and Sub-County.Vol. I. 2019. 38 p. Available from:* http://www.knbs.or.ke. 2019.
21. Bisanzio, D., et al., *Use of prospective hospital surveillance data to define spatiotemporal heterogeneity of malaria risk in coastal Kenya*. Malaria journal, 2015. **14**(1): p. 482.
22. WG., C., *Sampling Techniques. ,* ed. Third. 1977., New York: John Wiley & Sons, Inc. .
23. WHO, *Basic malaria microscopy. Geneva: World Health Organization*. 2010.
24. Nega, D., et al., *Prevalence and predictors of asymptomatic malaria parasitemia among pregnant women in the rural surroundings of Arbaminch Town, South Ethiopia*. PLoS One, 2015. **10**(4).
25. Brabin, B.J., *An analysis of malaria in pregnancy in Africa*. Bulletin of the World Health Organization, 1983. **61**(6): p. 1005.
26. Matangila, J.R., et al., *Asymptomatic Plasmodium falciparum infection is associated with anaemia in pregnancy and can be more cost-effectively detected by rapid diagnostic test than by microscopy in Kinshasa, Democratic Republic of the Congo*. Malaria journal, 2014. **13**(1): p. 132.
27. Bouyou-Akotet, M.K., et al., *Prevalence of Plasmodium falciparum infection in pregnant women in Gabon*. Malaria journal, 2003. **2**(1): p. 18.
28. Yakubu, D., N. Kamji, and A. Dawet, *Prevalence of Malaria Among Pregnant Women Attending Antenatal Care at Faith Alive Foundation, Jos, Plateau State, Nigeria*. Noble International Journal of Scientific Research, 2018. **2**(4): p. 19-26.
29. Ivoke, N., et al., *Falciparum Malaria Parasitaemia Among Pregnant Women Attending Antenatal Clinics in a Guinea-Savannah Zone, Southwestern Ebonyi State, Nigeria*. International Journal of Scientific and Engineering Research, 2013. **4**(9): p. 1876-1883.
30. Amoo, A., et al., *Malaria parasitaemia. It's association with bacteramia, haemoglobin genotype and ABO blood group system*. Research Journal of Medical Sciences, 2008. **2**: p. 137-141.
31. Dicko, A., et al., *Risk factors for malaria infection and anemia for pregnant women in the Sahel area of Bandiagara, Mali*. Acta Tropica, 2003. **89**(1): p. 17-23.
32. Ouma, P., et al., *Malaria and anaemia among pregnant women at first antenatal clinic visit in Kisumu, western Kenya*. Tropical Medicine & International Health, 2007. **12**(12): p. 1515-1523.
33. Enato, E., et al., *Plasmodium falciparum malaria in pregnancy: prevalence of peripheral parasitaemia, anaemia and malaria care-seeking behaviour among pregnant women attending two antenatal clinics in Edo State, Nigeria*. Journal of Obstetrics and Gynaecology, 2009. **29**(4): p. 301-306.
34. WHO. *Intermittent preventive treatment of malaria in pregnancy (IPTp),* . 2017 14 June 2020 [cited 2020 14th June]; Available from: http://www.who.int/malaria/areas/preventive therapies/ pregnancy/en/.
35. Agan, T., et al., *Prevalence of asymptomatic malaria parasitaemia*. Asian pacific journal of tropical medicine, 2010. **3**(1): p. 51-54.

36. Mwangi, M.N., *Safety and efficacy of iron supplementation in pregnant Kenyan women*. 2014: Wageningen University.
37. Okube, O.T., et al., *Prevalence and factors associated with anaemia among pregnant women attending antenatal clinic in the second and third trimesters at pumwani maternity hospital, Kenya.* Open Journal of Obstetrics and Gynecology, 2016. **6**(01): p. 16.
38. Zekarias, B., et al., *Prevalence of Anemia and its Associated Factors among Pregnant Women Attending Antenatal Care (ANC) In Mizan Tepi University Teaching Hospital, South West Ethiopia.* Health Science Journal, 2017. **11**(5): p. 1-8.
39. Melku, M., et al., *Prevalence and predictors of maternal anemia during pregnancy in Gondar, Northwest Ethiopia: an institutional based cross-sectional study.* Anemia, 2014. **2014**.
40. Saute, F., et al., *Malaria in pregnancy in rural Mozambique: the role of parity, submicroscopic and multiple Plasmodium falciparum infections.* Tropical Medicine & International Health, 2002. **7**(1): p. 19-28.
41. Glover-Amengor, M., W. Owusu, and B. Akanmori, *Determinants of anaemia in pregnancy in Sekyere West District, Ghana.* Ghana medical journal, 2005. **39**(3): p. 102.
42. Gedefaw, L., et al., *Anaemia and associated factors among pregnant women attending antenatal care clinic in Walayita Sodo town, Southern Ethiopia.* Ethiopian journal of health sciences, 2015. **25**(2): p. 155-164.

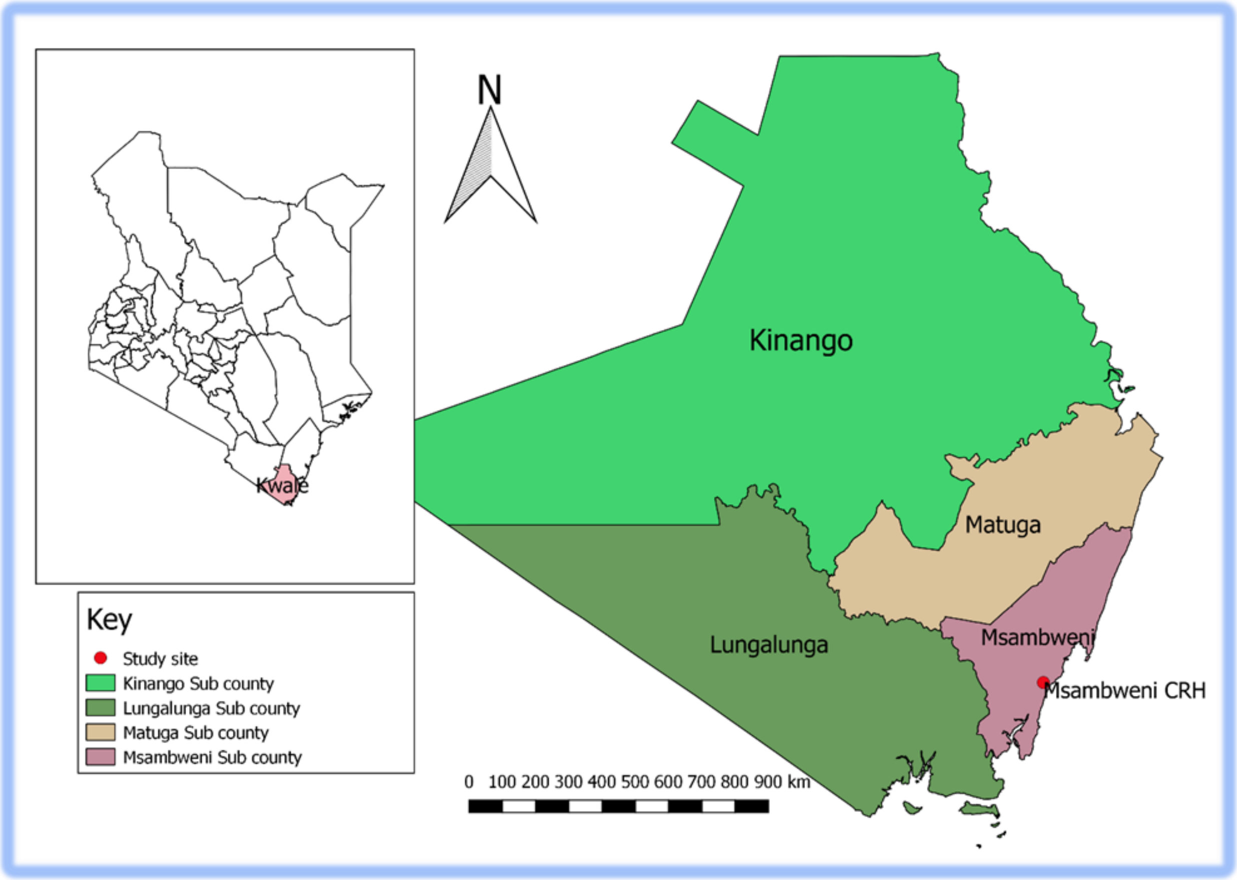

Figure 1: Map showing the study site – Kenyan Map. Counties Sampled (Map developed by Author using QGIS Version 2.18.10) with geographical data was obtained from https://africaopendata.org/dataset/kenya-counties-shapefile and data on geo - coordinates and category of health facility (Msambweni County Referral Hospital) ~~was~~ obtained from Kenya Master Health facility list http://kmhfl.health.go.ke/

Table 1: Socio demographic characteristics of women attending first antenatal care at Msambweni Hospital, Kwale County, Kenya.

Characteristics	n (%)
**Maternal age (n=308)**	
≤20	50 (16.2)
>20	258 (83.8)
**Education (n=301)**	
Had ≤8 years of formal schooling	218 (72.4)
Had > 8 years of formal schooling	83 (27.6)
**Residence (n=308)**	
Rural	275 (89.3)
Urban	33 (10.7)
**Marital status (n=307)**	
Married	267 (86.9)
Single	32 (10.4)
Divorced	6 (1.9)
Widowed	2 (0.7)
**Trimester (n=308)**	
First	29 (9.4)
Second	173 (56.2)
Third	106 (34.4)
**Gravidity (n=303)**	
Primigravidae	66 (21.8)
Secundigravidae	76 (25.1)
Multigravidae	161 (53.1)
**Gestational age in weeks (n=308)**	
<16	49 (15.9)
≥16	259 (84.1)
**Net ownership (n=308)**	
Yes	248 (80.5)
No	60 (19.5)
**Slept under bed net previous night (n =248)**	
Yes	231 (93.1)
No	17 (6.9)
**Frequency of sleeping under bed net (n=248)**	
Always	205 (82.7)
Sometimes	43 (17.3)
**Age of a bed net (in months) (n=246)**	
< 6 months	109 (44.3)
6-12 months	34 (13.8)
> 12 months	103 (41.9)

Table 2: Factors associated asymptomatic malaria parasitaemia in Pregnant Women, Kwale County, Kenya.

Potential factors	N	With Malaria n (%)	Crude POR (95% CI)	Adjusted POR (95% CI)
Maternal age (n=308)				
≤ 20	50	14 (28)	3.5 (1.66-7.26)	4.5 (1.71-12.01)
>20	258	26 (10.1)	1	1
Education (n=301)				
Had ≤ 8 years of formal schooling	218	27(12.4)	0.8 (0.40-1.74)	**
Had > 8 years of formal schooling	83	12(14.5)	1	**
Residence (n=308)				
Rural	275	36 (13.1)	1.1 (0.36-3.29)	**
Urban	33	4 (12.1)	1	**
Trimester (n=308)				
First/second	202	26 (12.9)	0.97 (0.48-1.95)	**
Third	106	14 (13.2)	1	**
Gravidity (n=303)				
Primigravidae/ Secundigravidae	145	23 (15.7)	1.6 (0.83-3.17)	**
Multigravidae	163	17 (10.4)	1	**
Gestational age in weeks (n=308)				
<16 weeks	49	7 (14.3)	1.14 (0.47-2.75)	**
≥16 weeks	259	33 (12.7)	1	**
Net ownership (n=308)		n=308		
No	60	13 (21.7)	2.3 (1.09-4.71)	**
Yes	248	27 (10.9)	1	**
Slept under bed net previous night (n=248)		n=288		
No	56	13(23.2)	2.3(1.14-5.01)	**
Yes	192	22 (11.5)	1	**
Frequency of sleeping under bed net (n=248)				
Sometimes	43	4 (9.3)	0.8 (0.28-2.62)	**
Always	205	22 (10.8)	1	**
Age of a bed net (in months) (n=246)				
> 12 months	103	15 (14.7)	1.9 (0.83-4.16)	**
≤12 months	143	12 (8.4)	1	**

CI, confidence interval, N, numbers, POR, prevalence odds ratio, aPOR=adjusted prevalence odds ratio

Table 3: Factors associated with Anemia in Pregnant Women, Kwale County, Kenya.				
Potential factors	N	With anemia (%)	Crude POR (95% CI)	Adjusted POR (95% CI)
**Maternal age (n=308)**				
≤ 20	50	34 (68.0)	1.3 (0.69-2.52)	**
>20	258	159 (61.6)	1	**
**Education (301)**				
Had ≤8 years of formal schooling	218	142 (65.1)	1.4 (0.85-2.39	**
Had > 8 years of formal schooling	83	47 (56.6)	1	**
**Residence (n=308)**				
Rural	275	175 (63.6)	1.5 (0.70-3.02)	**
Urban	33	18 (54.6)	1	**
**Trimester (n=308)**				
First	29	11 (5.7)	1	
Second	173	102 (52.9)	0.69(0.43-1.11)	**
Third	106	80(41.5)	5.0 (2.1-12.0)	**
**Gravidity (n=308)**				
Primigravidae	66	42 (21.8)	0.95(0.53-1.69)	**
Secundigravidae	79	49 (25.3)	0.91 (0.56- 1.45)	**
Multigravidae	163	102 (52.9)	1	
**Gestational age in weeks (n=308)**				
≥16 weeks	259	1741 (38.8)	3.2 (1.72-6.07)	3.3 (1.72-6.41)
<16 weeks	49	19 (67.2)	1	1
**Net ownership n=308**				
No	60	42 (30.0)	1.5 (0.82-2.75)	**
Yes	248	151 (70.0)	1	**
**Eating soil n=303**		n=303		
Yes	117	85(44.9)	2.1 (1.27-3.45)	2.0 (1.21-3.41)
No	186	104 (55.1)	1	1

CI, confidence interval, POR, prevalence odds ratio, aPOR=adjusted prevalence odds ratio

Table 4: Chi-square analysis of proportions with and without malaria parasitemia and anemia.

	Anemia						
Malaria parasitemia	Yes	No	Total	Crude POR	χ^2	p value	Adjusted POR
Yes	33 (17.1%)	7 (6.1%)	40	3.2 (1.36-7.46)	6.8	0.009	3.5 (1.46-8.60)
No	160(82.9%)	108 (93.9%)	268	ref			ref
Total	193	115	308				

POR, prevalence odds Ratio, Ref, reference

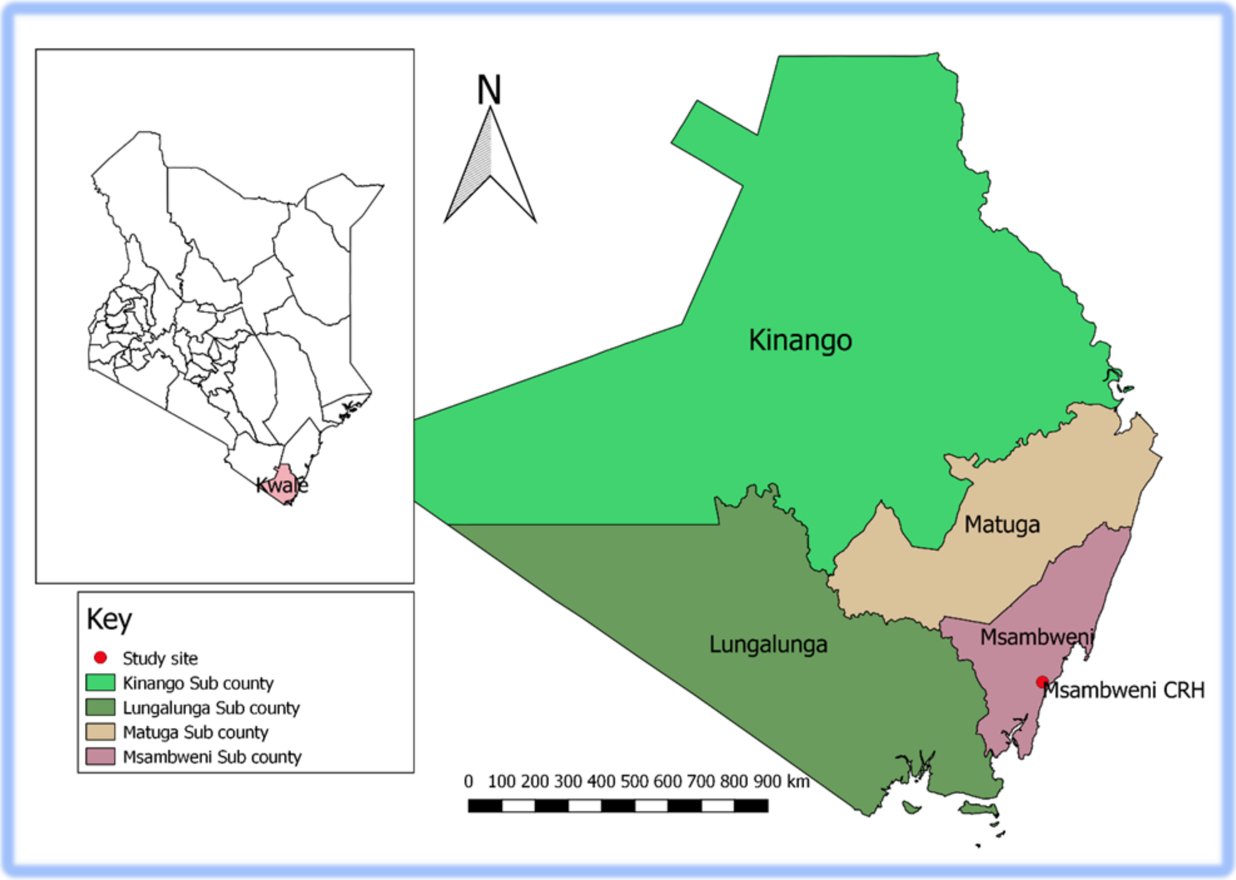

Figure 1: Map of the study area (Msambweni County Referral Hospital-MCRH)

Title: Prevalence and risk factors associated with asymptomatic Plasmodium *falciparum* infection and anemia among pregnant women at the first antenatal care visit: A hospital based cross-sectional study in Kwale County, Kenya.

Authors; Gibson Waweru[1, 2,] Jimmy Kihara[3], Elvis Oyugi[4], Victor Omballa[5], Hajara Busaidy[2], Victor Jeza[1]

Affiliations;

[1]Technical University of Mombasa, Kenya

[2]Departrtment of Health, Kwale County, Kenya

[3]Kenya Medical Research Institute, Kenya

[4]Kenya Field Epidemiology and Laboratory Training Program, Ministry of Health, Kenya

[5]Center for Global Health Research - Kenya Medical Research Institute, Nairobi, Kenya

Corresponding author
Mr. Gibson Nyamu, Msc
Head of the Kwale County Malaria Reference Laboratory,
Kwale County.
Tel: +254720633603
Fax: NA
Email: wawerugibson2015@gmail.com

Abstract

Background: Prevalence of both asymptomatic and symptomatic malaria in pregnancy (MiP) in Kenya ranges from 9% to 18%. We estimated the prevalence and factors associated with MiP and anemia in pregnancy (AiP) among asymptomatic women attending antenatal care (ANC) visits.

Methods: We performed a cross-sectional study among asymptomatic pregnant women attending ANC at Msambweni Hospital, between September 2018 and February 2019. Data was collected and analyzed in Epi Info 7. Descriptive statistics were calculated and we compared MiP and AiP in asymptomatic cases to those without either condition. Adjusted prevalence Odds ratios (aPOR) and 95% confidence intervals (CI) were calculated to identify factors associated with asymptomatic MiP and AiP.

Results: We interviewed 308 study participants; their mean age was 26.6 years (± 5.8 years), mean gestational age was 21.8 weeks (± 6.0 weeks), 173 (56.2%) were in the second trimester of pregnancy, ~~12.9 % (40/308) had MiP and 62.7 %~~ had AiP. Women who were aged ≤ 20 years had three times likelihood of developing MiP (aPOR= 3.1 CI: 1.3 - 7.35) compared to those aged >20 years old. The likelihood of AiP was higher among women with gestational age ≥ 16 weeks (aPOR=3.9, CI: 1.96 – 7.75), those with parasitemia (aPOR= 3.3, 95% CI: 1.31-8.18), those in third trimester of pregnancy (~~ aPOR~~= 2.6, 95% CI:1.40 - 4.96) and those who reported eating soil as a craving during pregnancy (aPOR=1.9, 95%CI:1.15 - 3.29).~~.~~

Conclusions: Majority of the women had asymptomatic AiP and MiP was observed in one tenth of all study participants. Asymptomatic MiP was associated with younger age while AiP was associated with gestational age, parasitemia, and soil consumption as a craving during pregnancy.

Key words: Anemia, Asymptomatic malaria, Pregnant, Kenya.

Introduction

The commonest plasmodium species that is known to cause malaria in pregnancy (MiP) in Africa is *Plasmodium falciparum* which can lead to anemia in pregnancy (AiP) [1]. The World Health Organization (WHO) in 2019, reported 11 million pregnant women were infected with plasmodium infection in Sub – Saharan Africa, resulting in 872 000 low birth weights [2]. In 2019, the Ministry of Health (MOH), Kenya, estimated MiP to be ~~89,324/1,42,896~~ (6.3%) among women attending their first antenatal care (ANC) visit [3]. In Kwale County, Kenya, MiP remains a public health concern with a total of 2,316 in 2019 [3]. Anemia in Pregnancy is a well-known risk factor for maternal death, stillbirths, low birth weights and infant prematurity [4-6]. Previous studies have reported associations between malaria with AiP [7, 8] while consumption of soil (geophagy) has been associated with AiP among African women [9, 10]. Besides plasmodium infections, other known causes of AiP include nutritional deficiencies, infectious diseases like HIV, parasitic infections like hookworm infestation, and hemoglobinopathies [4, 10].

Malaria in Pregnancy constitutes a major risk to the mother, fetus, and neonates including stillbirths, spontaneous abortion, premature delivery, maternal anemia, and low birth weight [2, 11]. Due to physiological and immunological changes, pregnant women have been found to have a higher risk of malaria compared to non-pregnant women living in areas of similar malaria endemicity [12]. Factors associated with MiP are less parity, low maternal education level, early gestational age, young maternal age, fewer previous pregnancies, non-ownership or infrequent use of bed nets and maternal unemployment [7, 11, 13-15].

Focused antenatal care is a package given to pregnant women which entails giving prophylaxis and treatment for anemia and malaria among other services, where the AiP is prevented by providing nutritional counseling including iron supplements, and treating cases of AiP [16]. Interventions aimed at prevention and control of MiP adopted by the MOH– Kenya are; Intermittent Preventive Treatment (IPTp) of MiP, with Sulfadoxine Pyrimethamine (SP) given after 12 weeks gestational period done four weeks apart until the pregnant woman delivers. In areas with high malaria transmission such as Western, Nyanza, and Coast regions, Long-lasting Insecticidal Nets (LLINs) are provided at the ANC during the first contact [17]. Kenya Malaria Indicator Survey (KMIS) 2015 reported two folds increase in the prevalence of malaria in the coastal region compared to KMIS 2010 [18]. In high-transmission regions like coastal regions in Kenya, where levels of acquired immunity tend to be high P. *falciparum* infection is usually asymptomatic in pregnancy. However, parasites may be present in the placenta and contribute to AiP even in the absence of documented peripheral parasitaemia [19]. Therefore, elimination of malaria is highly unlikely if diagnostic strategies do not include asymptomatic patients, because they will remain a reservoir of parasites contributing to the spread of the disease from one malaria season to the next. Management and control of MiP and AiP are enhanced by the availability of local prevalence statistics, which is not adequately provided in Kwale County except the data collected in passive surveillance according to records in the County. It is from this background that we carried out this study to estimate the prevalence of asymptomatic MiP and AiP and identify the associated factors among women attending their first ANC visit at the largest referral health facility in Kwale County, Kenya.

Methods

Study location

Kwale County is one of six counties in the coastal region of Kenya covering an area of 8,270.3 km^2 with a population of 866,820 people [20]. The inhabitants are predominantly Muslim, from the Mijikenda tribe, and practice subsistence farming and smallholder animal husbandry. The weather is hot and humid with two rainy seasons: long rains from April to June and short rains from October through November. The incidence of malaria increases during the long rainy seasons [21].

Msambweni County Referral Hospital (MCRH) is the main referral health facility in Kwale County (Figure 1). The hospital has 155 inpatient beds and189 healthcare workers. Four nurses work at the Mother-Child Health department, who attend on average 125 mothers each month [3].

Study design

We conducted across-sectional study to determine the prevalence of asymptomatic MiP and AiP.

Study population

Pregnant women attending their first ANC visit at MCRH between September 2018 and February 2019.

Inclusion criteria and exclusion criteria

Pregnant women seeking their first ANC were included in the study, especially those with no symptoms of malaria as per clinical assessment (i.e. no fever (temperature >37.5°C), chills, rigor, nausea, vomiting, headache, anorexia, or joint/muscle pains).

We excluded pregnant women who had taken anti-malarial drugs within the past two weeks, antipyretics in case they had fever and those receiving micronutrient.

Definitions of terms

- Asymptomatic malaria was defined as the presence in the peripheral blood of asexual blood stage of *Plasmodium*, irrespective of species but has no symptoms of malaria per clinical assessment and has not taken antipyretics within 48 hours and anti-malarials within 14 days.
- A young age was defined as age ≤ 20 years
- Anemia was defined as a hemoglobin < 11 g/dl while mild anemia (10 - 10.9g/dl), moderate anemia (7 - 9.9g/dl) and severe anemia (<7g/dl)
- First **trimester** was defined as; from **week** 1 to the end of **week** 12 while the second **trimester** as from **week** 13 to the end of **week** 26 and the third **trimester** as from **week** 27 to the end of the pregnancy

Sample size determination

Cochran's formula [22] was used to calculate the sample size required to estimate the prevalence of asymptomatic malaria in pregnant women attending their first ANC visit.

Assumptions made were:

- Two-sided significance level defined at 5%
- Power or percentage of detection defined at 80%
- The prevalence of asymptomatic MiP to be 24% [7].
- We adjusted by 10% to cater for those who refused to be enrolled. We calculated the desired sample size as 308 participants.

Sampling procedures

Systematic random sampling method was used to select study participants. Our sampling interval was based on the daily entries in the mother-child health (MCH) register from September 2018

to February 2019. The sampling started by selecting a participant from the daily entries list at random using a table of random numbers and then every k^{th} participant in the frame was selected. A selection interval *(k)* was determined by dividing the total daily entry listed in order to get the number of the participants required per day. If a randomly-selected participant was not eligible for an interview or refused to be part of the study, the next eligible participant on the list was selected. We sampled our study participants until we arrived at our desired sample size of 308.

Following signing of informed consent, the study participants were explained that those who tested positive for malaria and have anemia would benefit by being treated as per the guidelines of malaria and anemia in pregnancy at no extra cost.

Data collection

In-person interviews were conducted using a pre-tested structured questionnaire. The questionnaire was developed in English, and translated to Swahili for non-English speaking respondents.

Variables collected were;

- **Socio-demographic characteristics;** mother's age, education level, marital status and occupation
- **Obstetrics variables;** gravidity, parity, trimesters, and gestational age in weeks.
- **Clinical history variables;** history of fever in the last 48 hours and taken anti pyretic drugs, whether the client has taken antimalarial drugs within the last 2 weeks, tendency of geophagy, and whether or not on iron supplements.

Laboratory methods

Hemoglobin testing

The index finger was cleaned using 70% isopropyl alcohol and pricked using a sterile lancet by well-trained laboratory technicians. The first drop was wiped away using sterile cotton wool, and then the finger was gently squeezed to obtain approximately 30µl drop of blood onto a micro-cuvette and subsequently into a portable heme-analyzer (Hem cue Hb 301, Hemo Cue AB 16, Sweden). To determine anemia status, Hb measurement was obtained within 45 seconds and reported in grams per deciliters (gm/dl).

Malaria testing

Thick and thin blood films were prepared. Absolute methanol was used to fix thin films and Giemsa stain (3%) staining for 30 minutes. The slides were then rinsed with distilled water and air-dried at room temperature. Slides were then viewed under the microscope using 100x objectives on immersion oil. No Parasite Found (NPF) was reported after 100 fields were examined and no malaria parasites observed.

Thick films were examined to determine the presence of asexual malaria parasites, quantification of malaria parasites was done by enumerating asexual malaria parasites against 200 White blood cells (WBC). Then, Parasite densities (parasite per microliter of blood) were determined at after multiplying with an assumed WBC count of 8.0_109/l, with the product of numbers of malaria parasites divided by 200 WBC [23]. Also, the speciation of the *Plasmodium* parasites was done.

Quality Control

Quality of Giemsa stain was maintained by testing known positives slides. An independent qualified parasitologist examined 10% of both positives and negatives slides which were randomly selected and any variation were corrected before final reporting. Quality control of our hematology analyzer (HemocueHb 301, Hemo Cue AB 16, Sweden) was performed per as the

manufacturer instructions, by analyzing dried samples with known Hb levels before testing participant samples.

Data management and analysis

Data were entered, cleaned, and rechecked using MS Excel 2013. Data were analyzed using Epi Info 7. The following descriptive statistics were calculated: frequencies and proportions for categorical variables, and measures of central tendency (mean, median, and mode) and dispersion (range, interquartile range, and standard deviation) for continuous variables.

We tested the relationship between a variety of predictor variables, including socio-demographic factors and clinical history, and malaria status as the outcome variable, comparing participants who tested malaria positive with those who tested negative. We also compared participants who had anemia to those who did not. Both crude prevalence ratio (cPOR) and adjusted prevalence odds ratio (aPOR), and their 95% confidence intervals were calculated. Variables with $p < 0.05$ were considered statistically significant. Variables with p-values ≤ 0.20 were included in a logistic regression model using a backward stepwise elimination method to identify independently associated factors.

Ethics approval and consent to participate

We sought written, informed consent from each participant before interviewing and finger pricking for malaria blood slides and Hemoglobin level analysis. Permission was granted to conduct the study by Kwale Department of Health and Msambweni Hospital Director. Ethical clearance was obtained from the Pwani University – Ethical Review Committee (ERC/MSc/021/2018).

Results

Socio-demographic characteristics of respondents

A total of 308 respondents were interviewed. Their mean age was 26.6 years (± 5.8 years) , 267 (86.9%) were married, 83 (26.9 %) had > 8 years of formal schooling, and among them, 29 (9.4%) were formally employed. The mean gestational age was 21.8 weeks (± 6.0 weeks), 29 (9.4%) were in first trimester, 173 956.2%) were in second trimester and 106 (34.4%) were in third trimester of pregnancy.. Those who were primi-gravidae were 66 (21.4%), second-gravidae were 79 (25.7%) and multi-gravidae were 163 (52.9%) (Table1).

Among the participants, 248 (80.5%) owned bed nets (treated or untreated). Of these, 109 (44.3%) had used a bed net for less than 6 months, 34 (13.8%) had used a bed net for 6 - 12 months, and 103 (41.8%) had used a bed net for > 12 months. In terms of bed net usage in the current pregnancy, 231 (93.2%) reported having slept under a bed net the previous night while 205 (82.7%) reported always sleeping under a bed net, and 43(17.3%) reported sometimes sleeping under a bed net (Table 1).

Prevalence and factors associated with asymptomatic malaria

Malaria positivity among the 308 study participants was 12.9% (40/308) and the geometric mean parasite count was 3738 parasites per microliter of blood; 35 (87.5%) tested positive for *Plasmodium falciparum* , 3 (7.5%) *Plasmodium malariae* and 2 (5.0%) *Plasmodium ovale* . In regard to gestational trimesters with plasmodium infections, those in the first trimester were 2/29 (6.9%), second trimester 24/173 (13.9%) and those in the third trimester were 14/106 (13.2%).

The odds of asymptomatic MiP was higher in women who were aged ≤ 20 years (cPOR= 3.5, 95% CI= 1.65 – 7.23), women who did not own bed nets (cPOR= 2.3, 95% CI 1.08 – 4.69) and

women who owned bed nets but did not sleep under a bed net the night before the interview (cPOR= 2.4, 95% CI 1.14 – 5.03). After logistic regression analysis, asymptomatic MiP was independently associated with being age ≤ 20 years (aPOR= 4.5 (1.71– 12.01) compared with those aged >20 years (Table 2).

Prevalence and factors associated with anemia

Anemia was reported in 193 (62.7%) participants, and the mean Hb was 9.6 mg/dl (± 1.3mg/dl); 96 (49.7%) had moderate anemia, 90 (46.6%) had mild anemia and seven (3.6%) had severe anemia. Among those with severe anemia four had malaria, moderate anemia 15/95 (15.8%) and mild anemia was 14/90 (15.6%). Geophagy was reported by 117 (38.6%) participants. Those with a gestational age of ≥ 16 weeks had greater odds of AiP, cPOR= 3.2 (1.72 – 6.07) compared to those with gestational age <16 weeks. Those who reported eating soil had greater odds of AiP, cPOR= 2.1 (1.27 – 3.45) compared with those who did not report eating soil. Following logistic regression analysis, AiP was independently associated with gestational age ≥ 16 weeks of gestational period had (aPOR; 3.3, 95% CI: 1.72– 6.41), and those who reported eating soil (aPOR 2.0, 95% CI: 1.21– 3.41) Table 3.

Pregnant women with malaria parasitemia were three times more likely to have anemia compared to those without malaria parasitemia (χ^2=2.79, P-value=0.005, (aPOR; 3.5, 95% CI: 1.21– 8.60) (Table 4).

Discussion

Numerous studies have shown that anemia and malaria contribute to morbidity and mortality among pregnant women. In this study, we found that one in eight women had asymptomatic MiP and more than half of the women had AiP. Asymptomatic MiP was associated with young age ($\leq$ 20 years). Anemia prevalence was also associated with pregnant women who reported eating soil, were in their first and third trimesters of pregnancy, and had *P. falciparum* infections.

The prevalence of asymptomatic MiP in our study population was 12.9%, which is similar to the prevalence among pregnant women in Ethiopia (9.1%) [24] but lower than the prevalence found in Burkina Faso (24%) among pregnant women [7]. In this study, we included only pregnant women seeking ANC services for the first time in their current pregnancy and majority of study participants (82.7%) reported to always using bednet, whereas the study in Burkina Faso included pregnant women seeking ANC services at any point in their pregnancies. These may be factors that lead in the difference between these two reported prevalence.

In this study, we found that the odds of MiP among young women ($\leq$ 20 years of age) were greater than the odds of MiP among older women. This may reflect continuing development of malarial immunity [14, 25, 26]. Contrast to our findings, a study in Gabon showed that there is no significant difference was found between younger and older women [27].

The current study reports higher proportion of *P. falciparum* infections in pregnant women who were both in second and third trimesters and less proportion in the first trimester. Studies in Nigeria have also reported high malaria prevalence in pregnant women who were in their second trimesters [28-30]. Another study in Mali reported pregnant women in their first trimester were two times more likely to get malaria compared to the third trimester [31]. In contrast, our study reported less proportion of pregnant women in their first trimester was not associated with

malaria. This was probably due to the small number of pregnant women among this group. With an increase in the number of pregnant women in their first trimester, there is the possibility that there could be changes from the present results.

This study is showing highest proportional of Plasmodium infections was observed among the primi-gravidae (19.7%) followed by secundi-gravidae (12.7.7%) and multi-gravidae (10.4 %) with parasitaemia declining with increasing gravidity. These results are consistent with previous studies which found *Plasmodium* infections are more common in primi-gravidae women compared to multi-gravidae women [7, 29, 32]. The reason for the present result of gravidae-associated predisposition to *P. falciparum* infections may be due to the fact that adults who live in malaria-endemic regions generally have some acquired immunity to malaria infection. This acquired immunity diminishes significantly in pregnancy particularly in primi-gravidae. It has also been suggested by various authors that the early onset of antibody response in multi-gravidae and the delayed antibody production in primi-gravidae may be responsible for the gravidity-dependent and differential prevalence of *falciparum* malaria among pregnant women [19, 33].

Intervention measures for first visit pregnant women at antenatal clinic for malaria in pregnancy are; IPTp and provision of LLINs among others [17]. Although the role of IPTp is known to reduce maternal malaria episodes and improve pregnancy outcomes [34], the current study did not include pregnant women who had taken IPTp hence we could not ascertain the role played by IPTp. Our study evaluated through interviewing study participants on bed net ownership and usage. Majority of our respondents owned, slept under a bed net and almost all participants reported sleeping under a bed net the previous night. The KMIS, 2015, reported similar high rates of bed net ownership and use in pregnant women living in malaria-endemic zones, with

83.7% of pregnant women reporting sleeping under a bed net the night before they were interviewed [18]. Bed net ownership and usage have been reported in several studies to be protective against malaria infections [26, 35]. Our observed high rate of bed net ownership may be the result of a Kenyan national malaria policy which stipulates that pregnant women should routinely be issued with bed nets at their first ANC visits. In addition, Kenya's Malaria Control Program recently conducted a mass net distribution campaign, and all households were issued bed nets in Kwale County. Bed nets ownership have shown to be protective for malaria [36], in this study those pregnant women reported had no bed nets had 90 % higher odds of asymptomatic MiP compared to those who owned bed nets, though was not statistically significant.

The study reported more than half of the respondents (60%) had AiP. The etiology of anemia is variable and potentially multi-factorial, and thus several underlying morbid and co-morbid conditions may contribute to the prevalence of anemia. Similar to our findings, one study conducted in the Pumwani maternity hospital in Nairobi, Kenya reported an AiP prevalence of 57% [37], and another study in southern Ethiopia reported an AiP prevalence of 60% [34]. Lower prevalence of AiP have also been reported in southwest Ethiopia (23.5%) and northwest Ethiopia (16.5%) [38, 39].

The present study found that a significant number of women with asymptomatic MiP had AiP. Malaria in pregnancy is known to cause AiP, this association has also been reported in other studies in sub-Saharan Africa [7, 8].

We also found high reported rates of eating soil (geophagy), consistent with other studies that have found high rates of geophagy among pregnant African women as well as associations between geophagy and anemia [9, 10].

Our results indicate that, anemia is more common among women in their third trimester than women in their first trimester, similar to findings reported in other studies [40, 41]. Hemoglobin decreases through to the end of the third-trimester. Anemia is a function of plasma volume and red cell mass; both of which increase during pregnancy; but the increase in plasma volume is proportionately greater than the increase in red cell mass [42]. This might be due the fact that increase in trimester may cause reduction in maternal iron reserves. Also, anemia in the third trimester may be more likely due to higher nutrient demands of the fetus later in pregnancy.

Our study had several limitations. We collected data from September 2018 to February 2019, a period during which there is low malaria transmission. This could have resulted in the under estimation of the overall prevalence of asymptomatic MiP in our study area. A continuous monitoring throughout the year of MiP incidences will account for seasonality burden [21].

In addition, the study was hospital-based, excluding pregnant women who did not seek ANC services. While this may limit the generalizability of findings to the community, few women fail to seek antenatal care in our study area. Determination of factors associated with asymptomatic MiP and AiP in hospital-based studies provides a proxy indicator of predictors in the community of that particular facility when community-based surveys are not feasible.

Lastly, this study did not explore other factors that may contribute to anemia, including nutritional factors, soil-transmitted helminthes infection, and hereditary conditions such as sickle cell disease thus limiting our ability to assess the contribution of other causes of anemia during pregnancy. However, diagnosis of anemia was based on laboratory analysis and did not depend on clinical assessment as reported by other researchers.

Conclusion

Asymptomatic *Plasmodium* infections and anemia are common in women attending their first ANC visit at Msambweni County Referral Hospital in Kwale County. Most of the *Plasmodium* infections in this area are caused by *P. falciparum*. We did not observe a clear gravidity pattern of asymptomatic MiP although was associated with younger maternal age (≤20 years). This may be possible although unlikely that selection bias may have influenced these findings. Anemia in pregnancy was associated with *Plasmodium* infections, women who reported to have geophagy tendency and those who were their third trimester. In the study area, we recommend pregnant women should not delay their first ANC attendance, for less than 10% attended in their first trimester. All women of childbearing age should be included in measures to control Plasmodium infection and anemia by the National Malaria Control Program, reproductive health department and other non–state actors. Also, the reproductive health department should carry out health promotion and education on late adolescent and school going pregnancy for delay of sexual debut.

Acknowledgments

We would like to thank the Technical University of Mombasa, Kenya, for their support during the study. Special thanks go to study participants and to the Kwale County Department of Health authorities for their collaboration. We also thank the many Vector Borne Disease Control Unit (VBDCU) Laboratory officers, including Said Lipi, Joyce Bandika, Peter Siema, Charles Ng'ang'a, and Elton Mzungu for their dedication and meticulous microscopy. We extend a special thank you to nurse Wendy Losier, for her assistance with the consenting process and data collection. We also thank Dr. Robert Perry, Dr. Elizabeth Wanja, and Dr. Shama Cash-Goldwasser for their valuable insight and comments on the manuscript.

References

1. Alaku, I., A. Abdullahi, and H. Kana, *Epidemiology of Malaria Parasites Infection among pregnant women in some part of Nasarawa State, Nigeria*. Developing Country Studies, 2015. **5**(2).
2. WHO. *WORLD MALARIA REPORT*
2019 14 th June 2020 [cited 2020 14th June]; Available from: https://www.who.int/malaria/publications/world-malaria-report-2019/en/.
3. DHIS2, *District Health Information Systems*. 2019.
4. McClure, E.M., et al., *The association of parasitic infections in pregnancy and maternal and fetal anemia: a cohort study in coastal Kenya*. PLoS neglected tropical diseases, 2014. **8**(2).
5. Helmy, M.E., N.I. Elkhouly, and R.A. Ghalab, *Maternal anemia with pregnancy and its adverse effects*. Menoufia Medical Journal, 2018. **31**(1): p. 7.
6. Uneke, C., D. Duhlinska, and E. Igbinedion, *Prevalence and public-health significance of*

HIV infection and anaemia among pregnant women attending antenatal clinics in south-eastern Nigeria. Journal of health, population, and nutrition, 2007. **25**(3): p. 328.

7. Douamba, Z., et al., *Asymptomatic malaria correlates with anaemia in pregnant women at Ouagadougou, Burkina Faso*. BioMed Research International, 2012. **2012**.
8. Isah, A.Y., M.A. Amanabo, and B.A. Ekele, *Prevalence of malaria parasitemia amongst asymptomatic pregnant women attending a Nigerian teaching hospital*. Annals of African medicine, 2011. **10**(2).
9. Mathee, A., et al., *A cross-sectional analytical study of geophagia practices and blood metal concentrations in pregnant women in Johannesburg, South Africa*. South African Medical Journal, 2014. **104**(8): p. 568-573.
10. Stephen, G., et al., *Anaemia in pregnancy: prevalence, risk factors, and adverse*

perinatal outcomes in Northern Tanzania. Anemia, 2018. **2018**.

11. Gajida, A., Z. Iliyasu, and A. Zoakah, *Malaria among antenatal clients attending primary health care facilities in Kano state, Nigeria*. Annals of African medicine, 2010. **9**(3).
12. Tarning, J., et al., *Population pharmacokinetics of dihydroartemisinin and piperaquine in pregnant and nonpregnant women with uncomplicated malaria*. Antimicrobial agents and chemotherapy, 2012. **56**(4): p. 1997-2007.
13. Olubukola, A., A. Odunayo, and A. Adesina, *Anemia in pregnancy at two levels of health care in Ibadan, south west Nigeria*. Annals of African medicine, 2011. **10**(4).
14. Agomo, C.O. and W.A. Oyibo, *Factors associated with risk of malaria infection among pregnant women in Lagos, Nigeria*. Infectious diseases of poverty, 2013. **2**(1): p. 19.

15. Nega, D., et al., *Anemia associated with asymptomatic malaria among pregnant women in the rural surroundings of Arba Minch Town, South Ethiopia*. BMC research notes, 2015. **8**(1): p. 110.
16. MOH. *Focused Antenatal Care*,. 2015 11th [cited 2020 June]; Available from: https://www.researchgate.net/publication/241510221.
17. MOH. *Malaria in Pregnancy*. 2020 June [cited 2020 14th]; Available from: http://www.nmcp.or.ke/index.php/malaria-in-pregnancy.
18. MOH. *Kenya Malaria Indicator Survey*. 2015 14 th June 2020 [cited 2020 14th]; Available from: https://dhsprogram.com/pubs/pdf/MIS22/MIS22.pdf.
19. Desai, M., et al., *Epidemiology and burden of malaria in pregnancy*. The Lancet infectious diseases, 2007. **7**(2): p. 93-104.
20. KNBS, *Kenya Population and Housing Census Volume I : Population By County*

and Sub-County.Vol. I. 2019. 38 p. Available from: http://www.knbs.or.ke. 2019.
21. Bisanzio, D., et al., *Use of prospective hospital surveillance data to define spatiotemporal heterogeneity of malaria risk in coastal Kenya*. Malaria journal, 2015. **14**(1): p. 482.
22. WG., C., *Sampling Techniques.* , ed. Third. 1977., New York: John Wiley &
Sons, Inc. .
23. WHO, *Basic malaria microscopy. Geneva: World Health*
Organization. 2010.
24. Nega, D., et al., *Prevalence and predictors of asymptomatic malaria parasitemia among pregnant women in the rural surroundings of Arbaminch Town, South Ethiopia*. PLoS One, 2015. **10**(4).
25. Brabin, B.J., *An analysis of malaria in pregnancy in Africa*. Bulletin of the World Health Organization, 1983. **61**(6): p. 1005.

26. Matangila, J.R., et al., *Asymptomatic Plasmodium falciparum infection is associated with anaemia in pregnancy and can be more cost-effectively detected by rapid diagnostic test than by microscopy in Kinshasa, Democratic Republic of the Congo*. Malaria journal, 2014. **13**(1): p. 132.
27. Bouyou-Akotet, M.K., et al., *Prevalence of Plasmodium falciparum infection in pregnant women in Gabon*. Malaria journal, 2003. **2**(1): p. 18.
28. Yakubu, D., N. Kamji, and A. Dawet, *Prevalence of Malaria Among Pregnant Women Attending Antenatal Care at Faith Alive Foundation, Jos, Plateau State, Nigeria*. Noble International Journal of Scientific Research, 2018. **2**(4): p. 19-26.
29. Ivoke, N., et al., *Falciparum Malaria Parasitaemia Among Pregnant Women Attending Antenatal Clinics in a Guinea-Savannah Zone, Southwestern Ebonyi State, Nigeria*. International Journal of Scientific

and Engineering Research, 2013. **4**(9): p. 1876-1883.
30. Amoo, A., et al., *Malaria parasitaemia. It's association with bacteramia, haemoglobin genotype and ABO blood group system*. Research Journal of Medical Sciences, 2008. **2**: p. 137-141.
31. Dicko, A., et al., *Risk factors for malaria infection and anemia for pregnant women in the Sahel area of Bandiagara, Mali*. Acta Tropica, 2003. **89**(1): p. 17-23.
32. Ouma, P., et al., *Malaria and anaemia among pregnant women at first antenatal clinic visit in Kisumu, western Kenya*. Tropical Medicine & International Health, 2007. **12**(12): p. 1515-1523.
33. Enato, E., et al., *Plasmodium falciparum malaria in pregnancy: prevalence of peripheral parasitaemia, anaemia and malaria care-seeking behaviour among pregnant women attending two antenatal clinics in Edo State, Nigeria*. Journal of

Obstetrics and Gynaecology, 2009. **29**(4): p. 301-306.
34. WHO. *Intermittent preventive treatment of malaria in pregnancy (IPTp),* . 2017 14 June 2020 [cited 2020 14th June]; Available from: http://www.who.int/malaria/areas/preventive therapies/ pregnancy/en/.
35. Agan, T., et al., *Prevalence of asymptomatic malaria parasitaemia.* Asian pacific journal of tropical medicine, 2010. **3**(1): p. 51-54.
36. Mwangi, M.N., *Safety and efficacy of iron supplementation in pregnant Kenyan women*. 2014: Wageningen University.
37. Okube, O.T., et al., *Prevalence and factors associated with anaemia among pregnant women attending antenatal clinic in the second and third trimesters at pumwani maternity hospital, Kenya.* Open Journal of Obstetrics and Gynecology, 2016. **6**(01): p. 16.

38. Zekarias, B., et al., *Prevalence of Anemia and its Associated Factors among Pregnant Women Attending Antenatal Care (ANC) In Mizan Tepi University Teaching Hospital, South West Ethiopia*. Health Science Journal, 2017. **11**(5): p. 1-8.
39. Melku, M., et al., *Prevalence and predictors of maternal anemia during pregnancy in Gondar, Northwest Ethiopia: an institutional based cross-sectional study*. Anemia, 2014. **2014**.
40. Saute, F., et al., *Malaria in pregnancy in rural Mozambique: the role of parity, submicroscopic and multiple Plasmodium falciparum infections*. Tropical Medicine & International Health, 2002. **7**(1): p. 19-28.
41. Glover-Amengor, M., W. Owusu, and B. Akanmori, *Determinants of anaemia in pregnancy in Sekyere West District, Ghana*. Ghana medical journal, 2005. **39**(3): p. 102.
42. Gedefaw, L., et al., *Anaemia and associated factors among pregnant women attending antenatal care clinic in Walayita Sodo town,*

Southern Ethiopia. Ethiopian journal of health sciences, 2015. **25**(2): p. 155-164.

Corrections on reviews done for Reviewer #2

Prevalence and risk factors associated with asymptomatic Plasmodium *falciparum* infection and anemia among pregnant women at the first antenatal care visit: A hospital based cross-sectional study in Kwale County, Kenya.

	Comments /questions	Corrections/ responses
Concern 1	**Suggested title:** "Prevalence and risk factors associated with asymptomatic Plasmodium falciparum infection and anaemia among pregnant women at the first antenatal care visit: A hospital based cross-sectional study in Kwale County, Kenya. Use asymptomatic malaria is less specific	Plasmodium falciparum was the predominant species more than 80% hence we concur with the reviewer<br>Prevalence and risk factors associated with asymptomatic Plasmodium *falciparum* infection and anemia among pregnant women at the first antenatal care visit: A hospital based cross-sectional study in Kwale County, Kenya.
Concern 2	**Abbreviations:** Malaria in pregnancy (MiP and not MIP), Anaemia in pregnancy (AiP and not AIP)	Have corrected the whole manuscript where applicable MIP to MiP and AIP to AiP
Concern 3	**Abstract**:<br>a) Background sub-session: Prevalence of malaria in pregnancy, not … in pregnant	Corrected to read Prevalence of malaria in pregnancy, not … in pregnant
	b) Results sub-session: please specify what type of odds you used for AiP (crude or adjusted)	We have corrected the omission, the odds Ratio was Adjusted Odd Ratio
	**c) Key words:** important key words do not appear. Please use "asymptomatic malaria" instead of "parasitaemia" which could lead confusion as including symptomatic and asymptomatic cases	Have replaced "Parasitaemia", with "asymptomatic malaria"
Concern 4	**Introduction**<br>a. The authors should provide more detail on the current policies against malaria and anaemia	"We have included these paragraphs<br>The focused antenatal care is a package given to pregnant women which entails giving prophylaxis and treatment for anemia and malaria, among other services, where the AiP is prevented by

	during pregnancy used in Kenya, particularly IPTp administration and timeline, iron and folate supplementation? Who in charge of these measures (Government or pregnant women themselves)?	providing nutritional counseling including iron supplements, and treating cases of AiP [20].<br>An intervention aimed at prevention and control MiP adopted by the Ministry of Health (MOH) Kenya, are; Intermittent Preventive Treatment (IPTp) of MiP, with Sulfadoxine Pyrimethamine (SP) given after 12 weeks gestational period done four weeks apart until the pregnant woman delivers, in areas with high malaria transmission such as Western, Nyanza and Coast regions and Long-lasting Insecticidal Nets (LLINs) at the ANC in the first contact among others [21]
	Page 3, line 6: "Other factors associated with MiP are……" Use "Other" supposes that you have cited first factors which are not the case. Please rephrase the sentence to make it clear.	Besides plasmodium infections other factors known to cause AiP include nutritional deficiencies, infectious diseases like HIV, parasitic infections like hookworm infestation, and the hemoglobinopathies [4, 5].
	c. Page 3, line 7: The authors state that "high parity" is associated with malaria. What do you mean by high parity? Multigravidae women? If yes, I think it's a wording mistake as it's well-known that is the primigravidae who are higher risk of MiP, so 'less parity".	Corrected; Several studies has documented factors associated with MiP are women with less parity,
	d. The rational of the study is unclear as presented. Please, give more details explaining what the study brings for the scientific community which are not already known. The only fact that "factors contributing to MiP are not well-described in this part of Kenya" is not sufficient.	Kenya Malaria Indicator Survey (KMIS) 2015 reported 2 folds increase in the prevalence of malaria in the coastal region compared to KMIS 2010 [18]. There are a possibility plasmodium infections in healthy adults, including pregnant women, in moderate to high transmission areas rarely result in fever [19]. Therefore, elimination of malaria is highly unlikely if diagnostic strategies do not include asymptomatic patients, because they will remain a reservoir of parasites contributing to the spread of the disease from one malaria season to the next. Management and control of MiP and AiP are enhanced by the availability of local prevalence

		statistics, which is not adequately provided in Kwale County except the data collected in passive surveillance. It is in this background we carried out this study to estimate the prevalence of asymptomatic MiP and AiP and identify the associated factors among women attending their first ANC visit at the largest referral health facility in Kwale County.
	e. What is the most common species of malaria parasites in Kenya? I suppose "P. falciparum". Hence, it would interest to adjust the title of manuscript accordingly.	Has adjusted the title by including the "asymptomatic Plasmodium falciparum infection"
Concern 5	Methods<br>a) Ethical statement should be presented in the main text.	
	i) Did the pregnant women receive IPTp at the 1st ANC if they were eligible?	Yes, they did, as per the government of Kenya Policy in prevention and control of Malaria in Pregnancy. .http://www.nmcp.or.ke/index.php/malaria-in-pregnancy
	ii. Did the pregnant women with asymptomatic malaria receive curative treatment? What and how (uncomplicated and severe malaria)? If not, why? Same concerns regarding the anaemia, particularly severe cases?	Following informed consent, the study participants were explained whose test positive for malaria and will have anemia will benefit by being treated as per the guidelines of malaria and anemia in pregnancy with no extra cost.
	iii. Any written informed consent? Any ethical committee approval?	We sought written, informed consent from each participant before interviewing and finger pricking for malaria blood slides and Hemoglobin level

		analysis. Permission was granted to conduct the study by Kwale Department of Health and Msambweni Hospital Director. Ethical clearance was obtained from the Pwani University -Ethical Review Committee (ERC/MSc/021/2018).
	b. Study population: give more detail on the strategy of participant's selection	Sampling procedures<br>Systematic random sampling method was used to select study participants. Our sampling interval was based on the daily entries in the mother-child health (MCH) register from September 2018 to February 2019. The sampling started by selecting a participant from the daily entries list at random using a table of random numbers and then every k^{th} participant in the frame was selected. A selection interval *(k)* was determined by dividing the total daily entry listed in order to get the number of the participants required per day. If a randomly-selected participant was not eligible for an interview or refused to be part of the study, the next eligible participant on the list was selected. We sampled our study participants until we arrived at our desired sample size of 308.<br>Following informed consent, the study participants were explained whose test positive for malaria and anemia will benefit by being treated as per the guidelines of malaria and anemia in pregnancy with no extra cost.
	c. Did you consider among the symptoms of malaria the history of fever the past 48 h before the visit?<br><br>Exclusion criteria: Pregnant women who had taken antimalarial drugs within the past two weeks were excluded. What about the women who had taken	Asymptomatic malaria was defined as the presence in the peripheral blood of asexual blood stage of Plasmodium, irrespective of species but has no symptoms of malaria per clinical assessment (i.e. temperature $\leq 37.50^{C}$, chills, rigor, nausea, vomiting,

	fever drug? There is a risk to consider women asymptomatic while they just took fever drug the day before the visit.	headache, anorexia, or joint/muscle pains) and has<br><br>not taken antipyretics within 48 hours and<br><br>antimalarials within 14 days.
Concern 6	e. Sample size: The authors have considered a prevalence of MiP from a study in Burkina Faso (24%) while the malaria transmission is different to both countries.	True, we took the prevalence of MiP for Burkina Faso (24%). To our best of our knowledge by the time we were conceptualizing the protocol there was no study for asymptomatic MiP we could get hence we used the African study similar to ours to calculate the prevalence. Also, we thought the differences in malaria prevalence between Kenya and Burkina Faso are more likely related to malaria transmission intensity due to we have similar climate is characterized by 'long rains' (April–June) and 'short rains' (October–December) rainy seasons.
	f. Data collection: give more details on socio-demographic characteristics, obstetric and clinical history.	Variables collected included;<br>**Socio-demographic characteristics;** mother's age, education level, marital status and occupation.<br>**Obstetrics variables;** gravidity, parity, trimesters, and gestational age in weeks.<br>**Clinical history variables;** history of fever in the last 48 hours and taken anti pyretic drugs, whether the client has taken antimalarials drugs, tendency of geophagy, whether patient is on iron supplements
	How did you assess the LLIN use?	We depended on what the study participant reported which has been highlighted as a limitation but we used a trained nurse on the protocol to collect the data.
	Please define the different trimesters of pregnancy (1st, 2nd, 3rd)?	First **trimester** was defined as; from **week** 1 to the end of **week** 12 while the second **trimester** is from **week** 13 to the end of **week** 26 and the third **trimester** is from **week** 27 to the end of the pregnancy
	Give more details on how the gestational age was assessed?	We used fundal height and last monthly period to estimate the gestational age

Concern 7	g. Quality control: Please precise if the 10% of slides chosen was for all sides or positive slides.	An independent qualified parasitologist examined 10% of both positives and negatives slides which were randomly selected
Concern 8	h. Statistical analysis: What procedure did you use for variable selection in the final model (multivariate model)?	Variables with p-value ≤ 0.20 were included in a logistic regression model using a backward stepwise elimination method to identify independently associated factors.
Concern 9	7) Results<br><br>a. Table 1: The proportion of pregnant women in the first trimester at the 1st ANC visit was 9.4% while the authors found that the proportion of pregnant women with gestational age < 16 wg was 15.9%. Why this discrepancy when the first trimester finished at 15 wg.<br><br>b. Table 2:<br>i. Why did you keep in the final multivariate model, the variable gestational age even if not significant in bivariate analysis?<br>ii. In the same way, why did you keep in the multivariable model "slept under bed net previous night" and "frequency of sleeping under bed net". Both variables seems to be correlate.<br>iii. However, you drop out the variable "gravidity" which should be forced in the final model even if not significant because it's a well-know factors strongly	a) The difference is due to the definition of the trimesters that was used in this study. First **trimester** was defined as; from **week** 1 to the end of **week** 12 while the second **trimester** is from **week** 13 to the end of **week** 26 and the third **trimester** is from **week** 27 to the end of the pregnancy<br><br>b) Have reanalyzed the data (see table 2 and 3)<br><br>c) Similar to the answer b, have re- analyzed the data (Table 2) and pooled together the primigravidae and second gravidae in comparison with multigravidae but still was not significant

	associated with MiP. Furthermore, I would like to suggest to the authors to make an sensitivity analysis by pooling primigravidae and secundigravidae in comparison to multigravidae.<br><br>c. Prevalence and factors associated with latent malaria:<br>i. Please define latent malaria?<br>ii. What is the prevalence of asymptomatic malaria among pregnant women in the 1st, 2nd and 3rd trimesters at the first ANC visit?<br>iii. What are the proportion of different species of parasites (P.f.; P.o; P.v; P.m)<br>iv. Please define POR at the first time it use in the text.<br><br>d. Prevalence and factors associated with anaemia:<br>i. Among the 3.6% of severe anaemia, how many	we have used I have replaced latent malaria with asymptomatic malaria<br><br>Gestational trimesters with plasmodium infections, the first trimester were 2/29 (6.9%), second trimester 24/173 (13.9%) and third trimester were 14/106 (13.2%).<br><br>Plasmodium *falciparum* were 35 (87.5%), Plasmodium *malarie* 3 (7.5%) and Plasmodium *ovale* 2 (5.0%).<br>I has been defined as Prevalence Odds Ratio<br><br>Those who had severe anemia 4/7 (57.1%) had malaria, moderate anemia 15/95 (15.8%) and mild anemia was 14/90 (15.6%).
	Discussion<br>a. Regarding the factors associated to MiP:<br>i. The authors should also discuss what happens among women in the first trimester of pregnancy. We can observe that women are more at risk of infection than those in 2nd and 3rd trimester (19.7%	The current study reported a higher proportion of Plasmodium infections in pregnant women who were both in second and third trimesters and less proportion to the first trimester. Studies in Nigeria have reported high malaria prevalence in pregnant women who were in their second trimesters [27-29]. A study in Mali reported pregnant women in their first trimester were two times more likely to get malaria compared to the third trimester [30]. In contrast, our study reported less proportion of pregnant women in their first trimester and was not

	vs. 12.7% and 10.4%, respectively).	associated with malaria. This was probably due to the small number of pregnant women among this category. With an increase in the number of pregnant women in their first trimester, there is the possibility that there could be changes from the present results.
	ii. The only factor associated with MiP is young age (< 20 y). The authors should consider to check an interaction between age and gravidity as both are correlated. Hence, this could be explained by that young pregnant women are mostly primigravidae? This deserves a couple of sentence in the discussion.	There was no Interaction<br>In this study, the highest proportional of Plasmodium infections was observed among the primigravidae (19.7%): followed by secundigravidae (12.7.7%) and multigravidae (10.4 %) with parasitaemia declining with increasing gravidity. These results are consistent with previous reports which found plasmodium infections are more common in primigravidae women compared to multigravidae women [7, 28, 31]. The reason for the present result of gravidae-associated predisposition to P. *falciparum* infections may be due to the fact that adults who live in malaria-endemic regions generally have some acquired immunity to malaria infection due to immunoglobulin production stimulated by previous malaria infection. This acquired immunity diminishes significantly in pregnancy particularly in primigravidae. It has also been suggested by various authors that the early onset of antibody response in multigravidae and the delayed antibody production in primigravidae may be responsible for the gravidity-dependent and differential prevalence of *falciparum* malaria among pregnant women [19, 32].
	b. Regarding the factors associated to AiP: First and 3rd trimester are both associated with AiP. This could be also explained by the haemoglobin level variation due to physiopathology of the pregnancy. This should be included in the discussion	We found that anemia is more common among women in their third trimester than among women in their first trimester, similar to findings reported in other studies [39, 40]. Hemoglobin decreases until the end of the third-trimester. This might be due the fact that increase in trimester may cause reduction in maternal iron reserves. Anemia is a function of plasma volume and red cell mass; both of which increase during pregnancy; but the increase in plasma volume is proportionately greater than the increase in red cell mass [41]. Also, anemia in the third trimester may be more likely due to higher nutrient demands of the fetus later in pregnancy

	c. Study limitations: The authors have stated several limitations for the study. It is a good point. However, they have to explain how they have controlled this bias to ensure the validity of the study.	We collected data from September 2018 to February 2019, a period during which there is low malaria transmission. This could have resulted in the underestimation of the overall prevalence of asymptomatic MiP in our study area. A continuous monitoring throughout the year of MiP incidences will account for seasonality burden [21].<br>In addition, the study was hospital-based, excluding pregnant women who did not seek ANC services. While this may limit the generalizability of findings to the community, few women fail to seek antenatal care in our study area. Determination of factors associated with asymptomatic MiP and AiP in hospital based studies provides a proxy indicator of predictors in the community of that particular facility when community based surveys are not feasible.<br>Lastly, this study did not explore other factors that may contribute to anemia, including nutritional factors, soil-transmitted helminthes infection, and hereditary conditions such as sickle cell disease thus limiting our ability to assess the contribution of other causes of anemia during pregnancy. However, diagnosis of anemia was based on laboratory analysis and did not depend on clinical assessment as reported by other researchers.
Concern 10	9) Conclusion: The authors should revise their conclusion in order to highlight the originality of the study.	Asymptomatic Plasmodium infections and anemia are common in women attending their first ANC visit at Msambweni County Referral Hospital in Kwale County. Most of the Plasmodium infections in this area are caused by P. *falciparum*. Asymptomatic MiP was associated with younger maternal age (≤20 years). Anemia in pregnancy was associated with Plasmodium infections, women who reported to have geophagy tendency and those who were their third trimester. In the study area, we recommend pregnant women should not delay their first ANC attendance, for less than 10% attended in their first trimester. All women of childbearing age should be included in measures to control Plasmodium infection and anemia by the National Malaria Control Program, reproductive health department and other non – state actors should. Also, the reproductive health department should carry out

		health promotion and education on late adolescent and school going pregnancy for delay of sexual debut.

1. Thesis title: Prevalence and associated factors with asymptomatic Plasmodium *falciparum* infection and anemia among pregnant women at the first antenatal care visit: A hospital based cross-sectional study in Kwale County.

**Questions/Concerns**	**Comments**	**Corrections**
**Major comments**		
Concern 1	Abstract results: make it clear whether the results presented are adjusted odds ratios throughout.	Corrected done it is adjusted prevalence odds ratios that has been used throughout the abstract
Concern 2	Risk factors for AIP: The authors need to better explain how anaemia risk is apparently associated with BOTH first trimester, AND gestation $\geq$16 weeks. There are problems with results presentation in Table 3 and it seems they have got the reference and comparator groups switched for the gestation comparison. In addition, part of the results in the table are presented as percentages of women with risk factor who are anaemia (the table column heading) and part are presented as percentage of anaemic women who fall into each risk factor category (not what we want). Please redo this table and recheck your AORs and revise text accordingly.	Yes, I have reanalyzed the anemia data, by use of backward elimination method, there seemed an interaction between trimester's variable and gestational variable. By removing trimester variable for it had a bigger p value, gestational $\geq$16 weeks resulted to AOR; 3.3 (1.72-6.41). I have changed the comparison groups accordingly both in the table and in the text
Concern 3	. Discussion: differences in malaria prevalence between Kenya and Burkina Faso are more likely related to malaria transmission intensity, prevalence falls with gestation, and falls more in women receiving IPTp. (Related to this, please also discuss role of IPTp in controlling MIP in conclusions).	Interventions measures for first visit pregnant women at antenatal clinic for malaria in pregnancy are; IPTp and provision of LLINs among

		others [17]. Although the role of IPTp is known to reduce maternal malaria episodes and improve pregnancy outcomes [33], the current study did not include pregnant women who had taken antimalarials hence we could not ascertain the role played by IPTp and majority of our study participants (82.7%) in the current study reported to always using bednet, whereas the study in Burkina Faso included pregnant women seeking ANC services at any point in their pregnancies. This may be factors in the difference between these two reported prevalence.
Concern 4	Similarly, end P 11, haemodilution over pregnancy seems more likely to explain declining hb over pregnancy. If postulating dfetal needs please provide reference.	Anemia is a function of plasma volume and red cell mass; both of which increase during pregnancy; but the increase in plasma volume is proportionately greater than the increase in red

		cell mass [40]. Explanation has been given
**Minor comments**		
Concern 1	Abstract: line 1 "pregnancy". Results 3rd sentence rewrite " Women who had MIP were 12.9%". Last line of results: rewrite for clarity.	Corrected; pregnant to pregnancy<br>Rewritten; Women who had plasmodium infections were 12.9 % (40/308)
Concern 2	There were a lot of spacing issues in the pdf, e.g. words joined together, or words and brackets without spaces before them. Examples in abstract methods: odds ratios(OR) and confidence intervals(CI)- there are many others.	Attention to details in spacing has been addressed throughout the manuscript
Concern 3	Introduction 3rd sentence change "malaria with AIP and consumption of soil (geophagy)", needs different punctuation?	It has been re-written
Concern 4	. Next sentence: is this risk factors for MIP (parasitic infections? High gravidity is NOT associated with MIP) Or AIP?	It has been re-written
Concern 5	"In sub-Saharan Africa, MIP affects approximately 125 million pregnant women every year"- this is a major misreading of the cited reference.	It has been re-written
Concern 6	Top of P 5 "a cross sectional"	It has been re- written
Concern7	Inclusion/exclusion: "as well as those who"	It has been re- written

Concern 8<br><br>Concern 8	Sample size "desired level" of what was 5%?	Two-sided significance level<br><br>defined at 5%
Concern 9	Data management " between a variety…"	It has been re-written
Concern 10	Page 8: "prevalence and risk factors for malaria parasitemia". Latent malaria is not a recognised phrase. Next line remove "who".	Latent replaced by asymptomatic and re-written
Concern 11	Parasite counts are best expressed as geometric mean not median.	Geometric mean was 3738
Concern 12	. P 9 defines mild, moderate, severe anaemia.	Mild, moderate and severe anemia has been defined in definition of terms
Concern 13	Page 11 first paragraph contains multiple grammatical errors, please rewrite	It has been re looked and corrected accordingly
Concern 14	Third paragraph same page please rewrite.	It has been re looked and corrected accordingly
Concern 15	A lot of the references are incomplete (lacking volumes/pages), or not enough details are given to retrieve them, or have formatting issues. Refs 2, 5, 6 (what is MOH? Where can this be obtained- similar comments for some others), 7, 17-22, 33.	The references has been corrected and the links are provided where necessary
Concern 16	Table groups need editing to ensure<br><br>e.g. it is clear which group women<br><br>with 8 y school fall into. Same for<br><br>gestation. And make table 2 N	The table 2; corrected $\leq$ 8 year in schooling and gestational age in weeks $\geq$16<br><br>It has been expanded and fonts increased for readability

	column wider so numbers are readable.	
