# Supplementary material for: Prevalence and risk factors associated with asymptomatic Plasmodium falciparum infection and anemia among pregnant women at the first antenatal care visit: A hospital based cross-sectional study in Kwale County, Kenya
Source: PLoS One. 2020 Oct 8;15(10):e0239578. doi: 10.1371/journal.pone.0239578 (PMC7544053; doi:10.1371/journal.pone.0239578)
Supplement: Attachment — Submitted filename: Response to Reviewers #2_13_08_2020.docx.pdf [file pone.0239578.s003.pdf]

Reviewer #2: While the revised manuscript has improved, it still requires significant grammatical editing to improve its readability and clarity. Some concepts remain unaddressed for me.

Serial Number	Concerns/ questions	Response /(s)
Concern 1	a) In the first review, I have highlighted that the rational of the study is unclear. The authors stated that pregnant women are potential reservoir of P.f parasites; however, the IPTp is one the main strategy protected pregnant women and is administered regardless the malaria status of women. Hence, this should not be a major lack as the women will clean by IPTp. In addition, in their conclusion, the authors recommended taking into account women of reproductive age in malaria control program; but no specific strategy has been suggested. The authors should revise their manuscript to show the scientific add-value of their paper.	In the study area, women have been reported delaying in starting pre-natal care during pregnancy which may also contribute to delay interventions measures uptake like IPTp and LLINs usage hence they will remain a reservoir of parasites contributing to the spread of the disease from one malaria season to the next [20].<br>We recommend the hospital in conjunction with Kwale Department of health should organize for regular outreach to the community targeting pregnant women for health education should be provided to positively influence the knowledge and attitudes of pregnant women of child-bearing age to malaria and anemia, including early antenatal registration.
Concern2	b) For positive malaria management, the authors just précised that they used per the government of Kenya<br>Policy in prevention and control of Malaria in Pregnancy. But they should presented shortly how these cases are managed in the manuscript.	Participants who were found with malaria parasitemia were treated with Artemether-lumefantrine for those who were in their 2nd and 3rd trimesters while those who were in the 1st trimester were treated with quinine.

Concern 3	c) Informed consent administration: What is the process for illiterate women who did not write?	Where a participant was not able to write or sign we ensured a witness of her choice observes the consent process and signs, on the behave of the study participant
Concern 4	d) There is no flow chart diagram? How many pregnant women ineligible were excluded to reach the 308 pregnant women? And what are the reasons? Also compared the baseline characteristics of the pregnant women included and those who did not	See figure 2
Concern 5	e) How many participants have been excluded because of the following criteria: Intake of antipyretic drugs within 48h?	Three (3)
Concern 6	f) I have a concerns with the cut-off used to define 1st, 2nd and 3rd trimester. The authors define first trimester by using a gestational age < 12 wg instead of 14 completed wg, the cut-off usually used. Why?	Well, we classified trimesters as this article "Malaria and Anemia in Pregnant and Non-Pregnant Women of Child-Bearing Age at the University Hospital, Kumasi, Ghana", [22] as we are doing literature reviews
Concern 7	g) Two methods were used to measure the gestational age (fundal height and LMP); but there is no precision in what situation one or both are used?	Both fundal height and LMP were used to measure the gestational age, unless the study participant reported she could not recall her LMP, the fundal height was only method to assess the gestational age.
Concern 8	h) QC of positive and negative slides. What is done in case of discrepancy?	An independent qualified parasitologist examined 10% of both positives and negatives slides which were randomly selected and in the case of any disparity they were read

		by a third parasitologist and his results were deemed final.
Concern 9	i) The authors showed that the prevalence of malaria were 6.9%; 13.9% and 13.2% in the first, 2nd and 3rd trimester respectively? It is slightly amazing as results as the prevalence should decrease from first trimester to delivery because of preventive measures implemented. How the authors explained this result. this deserve a couple of sentences in the discussion session	Well, the women attending ANC in the 1st, 2nd, and 3rd trimester the prevalence is different because this was a one off examination.<br>The assumptions were no standard package of preventive measures implemented across all pregnant women for their were in the first visit at ANC. Nevertheless the probable explanation may be our study had a few numbers in the first trimester compared with 2nd and 3rd trimesters hence the higher prevalence.
Concern 10	j) The authors highlighted the IPTp started from the 12 wg. I'm not sure if this is the current WHO recommendation as the IPTp should started as soon as possible from the 2nd trimester.	Intermittent Preventive Treatment (IPTp) of MiP, with Sulfadoxine Pyrimethamine (SP) given after 12 weeks (line 73,74,75)
Concern 11	k) Number the page of manuscript.	Numbering have been done
