# Supplementary material for: Prevalence and risk factors associated with asymptomatic Plasmodium falciparum infection and anemia among pregnant women at the first antenatal care visit: A hospital based cross-sectional study in Kwale County, Kenya
Source: PLoS One. 2020 Oct 8;15(10):e0239578. doi: 10.1371/journal.pone.0239578 (PMC7544053; doi:10.1371/journal.pone.0239578)
Supplement: Attachment — Submitted filename: Response to Reviewers#1.docx [file pone.0239578.s001.docx]

**1. Thesis title:** Prevalence and associated factors with asymptomatic Plasmodium *falciparum* infection and anemia among pregnant women at the first antenatal care visit: A hospital based cross-sectional study in Kwale County.

| **Questions/Concerns** | **Comments** | **Corrections** |
| --- | --- | --- |
| **Major comments** | | |
| Concern 1 | Abstract results: make it clear whether the results presented are adjusted odds ratios throughout. | Corrected done it is adjusted prevalence odds ratios that has been used throughout the abstract |
| Concern 2 | Risk factors for AIP: The authors need to better explain how anaemia risk is apparently associated with BOTH first trimester, AND gestation ≥16 weeks. There are problems with results presentation in Table 3 and it seems they have got the reference and comparator groups switched for the gestation comparison. In addition, part of the results in the table are presented as percentages of women with risk factor who are anaemia (the table column heading) and part are presented as percentage of anaemic women who fall into each risk factor category (not what we want). Please redo this table and recheck your AORs and revise text accordingly. | Yes, I have reanalyzed the anemia data, by use of backward elimination method, there seemed an interaction between trimester’s variable and gestational variable. By removing trimester variable for it had a bigger p value, gestational ≥16 weeks resulted to AOR; 3.3 (1.72-6.41). I have changed the comparison groups accordingly both in the table and in the text |
| Concern 3 | . Discussion: differences in malaria prevalence between Kenya and Burkina Faso are more likely related to malaria transmission intensity, prevalence falls with gestation, and falls more in women receiving IPTp. (Related to this, please also discuss role of IPTp in controlling MIP in conclusions). | Interventions measures for first visit pregnant women at antenatal clinic for malaria in pregnancy are; IPTp and provision of LLINs among others [17]. Although the role of IPTp is known to reduce maternal malaria episodes and improve pregnancy outcomes [33], the current study did not include pregnant women who had taken antimalarials hence we could not ascertain the role played by IPTp and majority of our study participants (82.7%) in the current study reported to always using bednet, whereas the study in Burkina Faso included pregnant women seeking ANC services at any point in their pregnancies. This may be factors in the difference between these two reported prevalence. |
| Concern 4 | Similarly, end P 11, haemodilution over pregnancy seems more likely to explain declining hb over pregnancy. If postulating dfetal needs please provide reference. | Anemia is a function of plasma volume and red cell mass; both of which increase during pregnancy; but the increase in plasma volume is proportionately greater than the increase in red cell mass [40]. Explanation has been given |
| **Minor comments** | | |
| Concern 1 | Abstract: line 1 “pregnancy”. Results 3rd sentence rewrite “ Women who had MIP were 12.9%”. Last line of results: rewrite for clarity. | Corrected; pregnant to pregnancy  Rewritten; Women who had plasmodium infections were 12.9 % (40/308) |
| Concern 2 | There were a lot of spacing issues in the pdf, e.g. words joined together, or words and brackets without spaces before them. Examples in abstract methods: odds ratios(OR) and confidence intervals(CI)- there are many others. | Attention to details in spacing has been addressed throughout the manuscript |
| Concern 3 | Introduction 3rd sentence change “malaria with AIP and consumption of soil (geophagy)”, needs different punctuation? | It has been re-written |
| Concern 4 | . Next sentence: is this risk factors for MIP (parasitic infections? High gravidity is NOT associated with MIP) Or AIP? | It has been re-written |
| Concern 5 | “In sub-Saharan Africa, MIP affects approximately 125 million pregnant women every year”- this is a major misreading of the cited reference. | It has been re-written |
| Concern 6 | Top of P 5 “a cross sectional” | It has been re- written |
| Concern7 | Inclusion/exclusion: “as well as those who” | It has been re- written |
| Concern 8  Concern 8 | Sample size “desired level” of what was 5%? | Two-sided significance level defined at 5% |
| Concern 9 | Data management “ between a variety…” | It has been re-written |
| Concern 10 | Page 8: “prevalence and risk factors for malaria parasitemia”. Latent malaria is not a recognised phrase. Next line remove “who”. | Latent replaced by asymptomatic and re-written |
| Concern 11 | Parasite counts are best expressed as geometric mean not median. | Geometric mean was 3738 |
| Concern 12 | . P 9 defines mild, moderate, severe anaemia. | Mild, moderate and severe anemia has been defined in definition of terms |
| Concern 13 | Page 11 first paragraph contains multiple grammatical errors, please rewrite | It has been re looked and corrected accordingly |
| Concern 14 | Third paragraph same page please rewrite. | It has been re looked and corrected accordingly |
| Concern 15 | A lot of the references are incomplete (lacking volumes/pages), or not enough details are given to retrieve them, or have formatting issues. Refs 2, 5, 6 (what is MOH? Where can this be obtained- similar comments for some others), 7, 17-22, 33. | The references has been corrected and the links are provided where necessary |
| Concern 16 | Table groups need editing to ensure e.g. it is clear which group women with 8 y school fall into. Same for gestation. And make table 2 N column wider so numbers are readable. | The table 2; corrected ≤ 8 year in schooling and gestational age in weeks ≥16  It has been expanded and fonts increased for readability |
